# CRISPR screens identify cholesterol biosynthesis as a therapeutic target on stemness and drug resistance of colon cancer

**DOI:** 10.1038/s41388-021-01882-7

**Published:** 2021-10-07

**Authors:** Shanshan Gao, Fraser Soares, Shiyan Wang, Chi Chun Wong, Huarong Chen, Zhenjie Yang, Weixin Liu, Minnie Y. Y. Go, Musaddeque Ahmed, Yong Zeng, Catherine Adell O’Brien, Joseph J. Y. Sung, Housheng Hansen He, Jun Yu

**Affiliations:** 1 Institute of Digestive Disease and Department of Medicine and Therapeutics, State Key Laboratory of Digestive Disease, Li Ka Shing Institute of Health Sciences, CUHK Shenzhen Research Institute, The Chinese University of Hong Kong, Hong Kong, China; 2 Princess Margaret Cancer Centre, University Health Network, Ontario, ON Canada; 3 Department of Medical Biophysics, University of Toronto, Ontario, ON Canada

**Keywords:** Colorectal cancer, Target identification

## Abstract

Cancer stem cells (CSCs) are responsible for tumor progression, recurrence, and drug resistance. To identify genetic vulnerabilities of colon cancer, we performed targeted CRISPR dropout screens comprising 657 Drugbank targets and 317 epigenetic regulators on two patient-derived colon CSC-enriched spheroids. Next-generation sequencing of pooled genomic DNAs isolated from surviving cells yielded therapeutic candidates. We unraveled 44 essential genes for colon CSC-enriched spheroids propagation, including key cholesterol biosynthetic genes (HMGCR, FDPS, and GGPS1). Cholesterol biosynthesis was induced in colon cancer tissues, especially CSC-enriched spheroids. The genetic and pharmacological inhibition of HMGCR/FDPS impaired self-renewal capacity and tumorigenic potential of the spheroid models in vitro and in vivo. Mechanistically, HMGCR or FDPS depletion impaired cancer stemness characteristics by activating TGF-β signaling, which in turn downregulated expression of inhibitors of differentiation (ID) proteins, key regulators of cancer stemness. Cholesterol and geranylgeranyl diphosphate (GGPP) rescued the growth inhibitory and signaling effect of HMGCR/FDPS blockade, implying a direct role of these metabolites in modulating stemness. Finally, cholesterol biosynthesis inhibitors and 5-FU demonstrated antitumor synergy in colon CSC-enriched spheroids, tumor organoids, and xenografts. Taken together, our study unravels novel genetic vulnerabilities of colon CSC-enriched spheroids and suggests cholesterol biosynthesis as a potential target in conjunction with traditional chemotherapy for colon cancer treatment.

## Introduction

Colon cancer is one of the most commonly diagnosed malignancy worldwide. The current standard of care for patients with colon cancer is maximal surgical removal of tumor, followed by adjuvant chemotherapy or targeted therapy [1]. However, drug resistance and tumor recurrence represent major clinical challenges of colon cancer. CSCs, or so-called cancer-initiating cells, comprise a tiny fraction of a tumor, but they are highly tumorigenic cells essential for tumor maintenance and a root cause of therapy resistance and tumor relapse [2–5]. Therefore, targeting CSCs is an attractive approach to eradicate tumors and prevent tumor recurrence, in contrast to conventional therapies that mostly target bulk tumor cells [6, 7]. For colon cancer, CSCs have been isolated with non-uniform surface markers, including CD133 [2, 3], Lgr5 [8], EpCAM, CD44, CD166 [9], ALDH [10], and EphB2 [5]. CSCs from primary colon tumors can be enriched and maintained by the well-established three-dimensional (3D) spheroid culture system, which preserves the capacity to re-establish cellular hierarchy recapitulating that of parental tumor and patient-specific genotypic and epigenetic signatures [2, 9, 11, 12]. These in vitro CSC-enriched spheroid models are clinically relevant in terms of their intrinsic resistance to conventional drugs [13]. In this study, we aim to uncover genetic vulnerabilities of colon CSC-enriched spheroids with a novel high-throughput CRISPR dropout screen.

CRISPR/Cas9-based dropout screening has emerged as a powerful tool for systematic identification of essential/fitness genes governing cell proliferation and survival [14–17]. In this study, we constructed an Epi-Drug single-guide RNA (sgRNA) library comprising of focused druggable genome targeted by FDA-approved drugs, together with numerous epigenetic regulators. Our Epi-Drug library enables the rapid prioritization of existing drug targets and drug repositioning for novel indications. In two colon cancer patient-derived CSC-enriched spheroid models, we performed Epi-Drug CRISPR dropout screens and identified potential druggable targets that could synergize with conventional chemotherapy. Our data provide a rationale for the novel druggable targets in colon cancer, especially for cell subpopulations harboring stemness and drug resistance characteristics.

## Results

### Epi-Drug CRISPR dropout screens identify genetic vulnerabilities of colon CSC-enriched spheroids

**Fig. 1:**
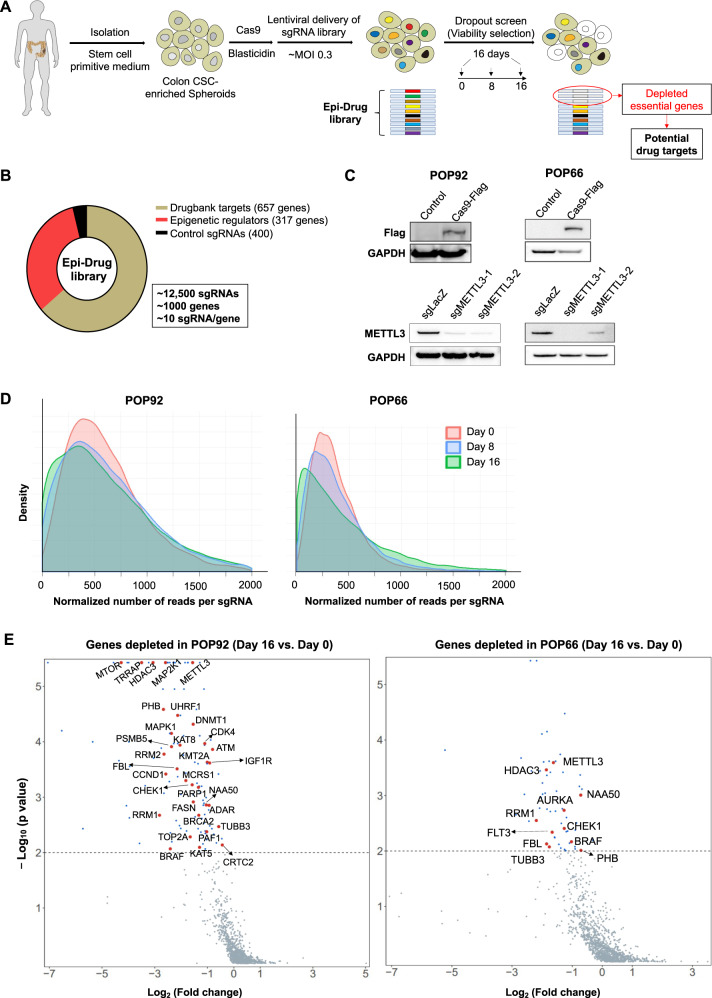
Targeted CRISPR-Cas9 dropout screens identify unique genetic vulnerabilities in patient-derived colon CSC-enriched spheroids. **A** Experimental workflow of the targeted CRISPR-Cas9 dropout screens. **B** Composition of the Epi-Drug sgRNA library. **C** Validation of the endonuclease activity of Cas9 protein with two sgRNAs targeting an individual gene METTL3 in two Cas9-expressing spheroids. **D** Distribution of sgRNA reads per gene at indicated time points post-transfection. **E** Volcano plots showing the genes significantly depleted in POP92 and POP66. Genes with *P* value < 0.01 were considered as essential for colon CSC-enriched spheroids propagation in vitro (threshold indicated by dashed line). Depleted genes were overlapped with a public CSC database, with matching genes highlighted in red. Source data of all genes are provided in Supplementary Tables S1 and S2.

In this study, we utilized two well-characterized, low-passage, patient-derived colon CSC-enriched spheroid lines, POP92 and POP66 (Fig. 1A) [11, 13]. Flow cytometry analysis confirmed >50% positivity in the stemness markers CD133 or CD44 in POP66/POP92 spheroids (Supplementary Fig. 1A). In addition, these stemness markers are functionally critical for maintenance of CSC-enriched spheroids, as CD133 knockout in spheroids inhibited cell proliferation, impaired sphere-formation ability, and induced cell differentiation (Supplementary Fig. 1B–D). To unravel druggable essential genes in these characterized CSC-enriched spheroids, we performed CRISPR/Cas9 dropout screening using our in-house Epi-Drug library comprising of over 12,500 sgRNAs targeting 657 Drugbank-based targets and 317 epigenetic regulators (Fig. 1B). We first generated stable Cas9-expressing spheroid models and confirmed the highly effective endonuclease activity with two sgRNAs targeting an individual gene METTL3 (Fig. 1C). Lentiviral Epi-Drug library was transduced into Cas9-expressing POP92 and POP66 spheroids at a low multiplicity of infection (MOI) of 0.3, and the abundance of individual sgRNAs at days 8 and 16 was quantified by next-generation sequencing compare to baseline pool. As shown in Fig. 1D, we observed the gradual depletion of sgRNAs over time in culture. Using the MAGeCK algorithm, we identified 121 and 59 essential genes depleted in POP92 and POP66 at a *P* value threshold of 0.01, respectively (Fig. 1E and Supplementary Tables S1, S2). We compared our list of essential genes to CSCdb, a database that includes 74 CSC markers and 1769 functional regulatory genes [18], and identified a significant overlap between the two datasets (Fig. 1E), implying that our screen successfully validated some well-known CSC regulators (e.g., mTOR, IGF1R, METTL3, HDAC3) [19–23].

### Cholesterol biosynthesis pathway is essential for colon CSC-enriched spheroids

**Fig. 2:**
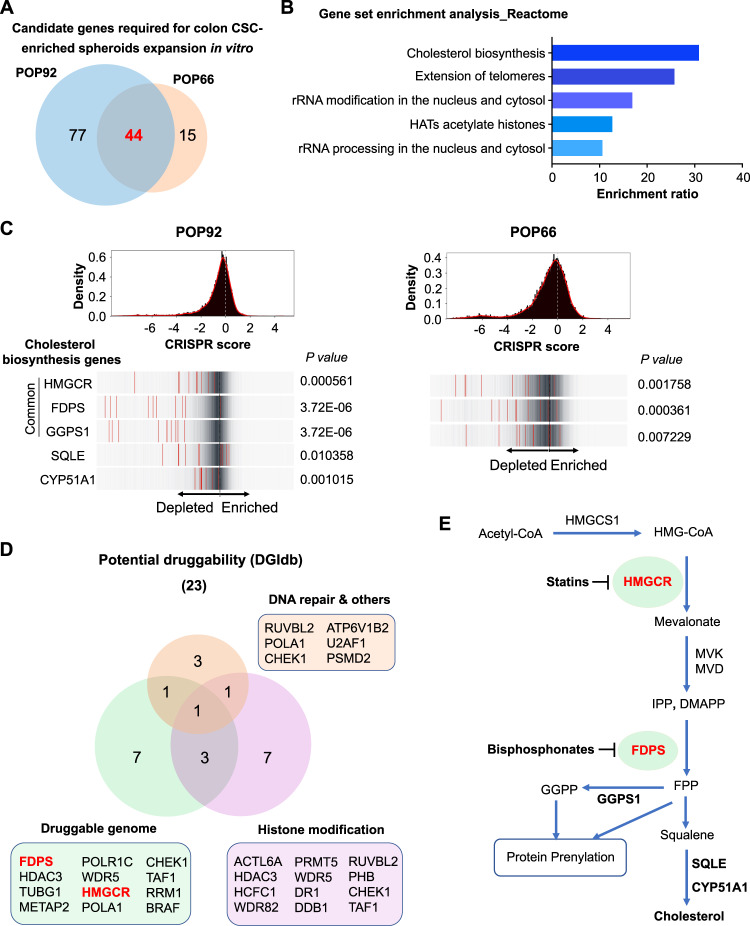
Analysis of essential genes for colon CSC-enriched spheroids expansion in vitro. **A** Venn diagram showing common essential genes in both CSC-enriched spheroid models, as listed in Table 1 and Supplementary Table S3. Genes are ranked by false discovery rate (FDR) in both models. **B** Gene set enrichment analysis of 44 common essential genes for both spheroid models. Pathways with FDR < 0.05 are shown. **C** Frequency histograms indicating distribution of CRISPR scores for all sgRNAs. Bottom panel shows distribution of individual sgRNAs (red lines) for genes involved in cholesterol biosynthesis pathway. **D** Drug Gene Interaction database (DGIdb) [24] categorization of the essential genes based on potential druggability defined by the DGIdb. Three categories are depicted. **E** Schematic diagram of the cholesterol biosynthesis pathway, also known as mevalonate pathway.

There were 44 common dropout essential genes in POP92 and POP66 (Fig. 2A, Table 1, and Supplementary Table S3), and pathway enrichment analysis revealed several significantly enriched terms, including cholesterol biosynthesis (Fig. 2B). Within the cholesterol biosynthesis pathway, HMGCR, FDPS, and GGPS1 were top-ranked essential genes for both POP92 and POP66, while SQLE and CYP51A1 were also important for POP92 (Fig. 2C). We next determined the “druggability” of the 44 common dropout genes using Drug Gene Interaction database and identified 23 genes that could be classified as “druggable” [24], including HMGCR and FDPS (Fig. 2D). HMGCR, known as HMG-CoA reductase, is the first rate-limiting enzyme for the mevalonate pathway that produces endogenous cholesterol (Fig. 2E). On the other hand, FDPS catalyzes the generation of farnesyl pyrophosphate (FPP) and GGPP, intermediates of cholesterol biosynthesis that serve as substrates for protein post-translational modification (Fig. 2E) [25]. Taken together, our findings indicate that cholesterol biosynthesis pathway is essential for the growth of colon CSC-enriched spheroids.

**Table 1:** Essential genes for both colon CSC-enriched spheroids propagation in vitro.

Rank	Gene symbol	Rank	Gene symbol	Rank	Gene symbol
1	ATP6V1B2	16	WDR5	31	HUWE1
2	WDR75	17	DR1	32	RTF1
3	ACTL6A	18	NOP2	33	POLA1
4	FDPS	19	DDB1	34	CHEK1
5	METTL3	20	RPL3	35	FBL
6	GGPS1	21	WDR74	36	TAF1
7	HDAC3	22	RUVBL2	37	RRM1
8	FTSJ3	23	RPL8	38	RUVBL1
9	TUBG1	24	PHB	39	TRMT112
10	HCFC1	25	HMGCR	40	U2AF1
11	WDR82	26	SUDS3	41	RBBP5
12	PRMT5	27	NAA50	42	TUBB3
13	METAP2	28	RPL4	43	PSMD2
14	POLR1C	29	DMAP1	44	BRAF
15	RPL19	30	RNF20		

### Cholesterol biosynthesis genes are overexpressed in human colon cancer and CSC-enriched spheroids

**Fig. 3:**
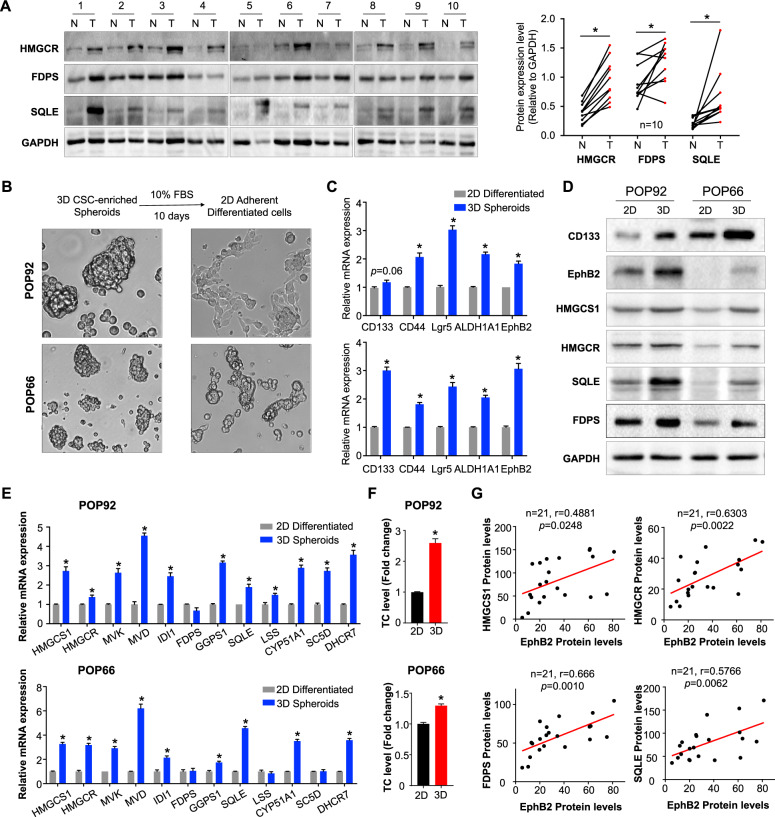
Cholesterol biosynthesis genes are upregulated in colon CSC-enriched spheroids and human primary colon cancer. **A** Cholesterol biosynthetic enzymes HMGCR, FDPS, and SQLE were evaluated in ten pairs of colon tumor (T) and matched non-tumor (N) specimens by western blot analysis. Quantitative densitometry analysis with ImageJ software (paired *t*-test). **B** Colon CSC-enriched spheroids were differentiated by adherent culture in 10% FBS-containing medium for 10 days. Representative images under the light microscope were shown. **C** CSC markers were determined by qRT-PCR in 3D colon CSC-enriched spheroids and their 2D differentiated counterparts. **D** CSC marker genes and cholesterol biosynthetic genes were investigated by western blot as indicated. **E** qRT-PCR analysis of cholesterol biosynthetic genes as indicated. **F** Total cholesterol (TC) levels were measured in colon CSC-enriched spheroids and their differentiated counterparts using the cholesterol/cholesteryl ester assay kit. **G** The correlations between cholesterol biosynthetic genes (HMGCR, HMGCS1, FDPS, SQLE) and intestinal stem cell marker EphB2 were evaluated by western blot in a cohort of 21 colon cancer patients. Error bar, mean ± SD. *P < 0.05 (*t*-test).

In light of our findings, we next investigated the potential role of cholesterol biosynthesis pathway in human colon cancer. We examined the expression of several key cholesterol biosynthetic enzymes (HMGCR, FDPS, and SQLE) in paired colon tumor and non-tumor tissues performed by western blot (Fig. 3A) and immunohistochemistry analysis (Supplementary Fig. 2A, B). Consistent with our hypothesis, all three genes are significantly upregulated in colon tumors compared to paired non-tumor tissues.

To investigate if cholesterol biosynthesis pathway is selectively upregulated in colon CSCs, we induced differentiation of CSC-enriched spheroids by culturing them in 2-dimensional adherent culture flasks with 10% FBS-containing medium for 10 days (Fig. 3B). Differentiation was validated by the reduced expression of stemness markers including CD133, CD44, Lgr5, ALDH1A1, and EphB2 (Fig. 3C, D). We next compared the expression of cholesterol biosynthetic enzymes in 3D CSC-enriched spheroids and their differentiated counterparts. Increased protein expression of several key genes including HMGCS1, HMGCR, SQLE, and FDPS was found in 3D CSC-enriched spheroids (Fig. 3D). In addition, marked and coordinated upregulation of most cholesterol biosynthesis enzymes were validated in 3D CSC-enriched spheroids by real-time PCR (Fig. 3E). Consistent with the augmented expression of cholesterol biosynthesis genes, total intracellular cholesterol content was significantly increased in both POP92 and POP66 spheroids as compared to their differentiated counterparts (Fig. 3F). These results indicate that cholesterol biosynthesis is markedly upregulated in colon CSC-enriched spheroids.

We then analyzed the correlation between cholesterol biosynthesis pathway and CSC markers in human colon cancer samples. We analyzed the correlation between stemness markers and cholesterol biosynthesis genes in TCGA colorectal cancer (COADREAD) cohort and revealed positive correlations between CSC markers (EphB2 and CD44) and cholesterol biosynthesis genes (HMGCR, HMGCS1, FDPS, and FDFT1) (Supplementary Fig. 3A). As EphB2 is highly correlated with cholesterol biosynthesis genes at mRNA level, we thus determined the correlation of cholesterol biosynthesis genes with EphB2 in a cohort of 21 human colon tumors by western blot and demonstrated that HMGCS1, HMGCR, FDPS, and SQLE positively correlated with EphB2 protein expression (Fig. 3G). In agreement with our data, analysis of the TCGA reverse phase protein array dataset revealed positive associations between several cholesterol biosynthetic enzymes and EphB2 (Supplementary Fig. 3B). Previous work has indicated EphB2 as a surface marker for stem-like tumor cells with robust tumor-initiating capacity and long-term self-renewal potential in human colorectal cancer [5]. Together, cholesterol biosynthesis activation is associated with cancer stemness traits in colon cancer.

### Genetic or pharmacological blockade of cholesterol biosynthesis impairs self-renewal and tumorigenic potential of CSC-enriched spheroids

**Fig. 4:**
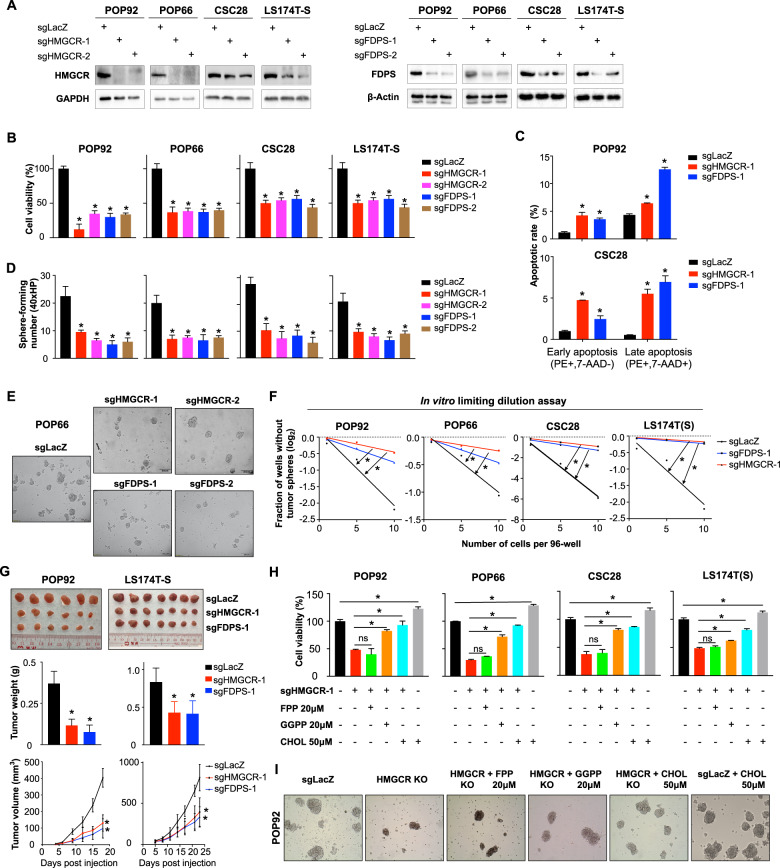
Genetic knockout of cholesterol biosynthetic enzymes impairs self-renewal and tumorigenic potential of colon CSC-enriched spheroids. **A** Colon CSC-enriched spheroids were transduced with sgRNAs targeting HMGCR or FDPS, followed by puromycin selection for 6 days. Knockout efficacy was evaluated by western blot. **B** Cell viability, **C** cell apoptosis, and **D** sphere-formation assays of colon CSC-enriched spheroids transduced with sgRNAs targeting indicated genes as compared to control. **E** Representative images of POP66 spheres treated as indicated. **F** In vitro limiting dilution assays of colon CSC-enriched spheroids after depletion of HMGCR or FDPS. **G** Representative images of excised tumors from mice injected with POP92 or LS174T-S spheroids after HMGCR or FDPS knockout. Tumor weight and tumor volume were measured. **H** Rescue assay using cholesterol biosynthesis pathway metabolites (FPP, GGPP, cholesterol) in HMGCR-depleted CSC-enriched spheroids. **I** Representative images of POP92 spheres treated as indicated. Error bar, mean ± SD. ns not significant, *P < 0.05 (*t*-test for **B**, **C**, **G**, one-way ANOVA for **H**).

To validate the functional importance of the cholesterol biosynthesis pathway in colon CSC-enriched spheroids, we evaluated the effect of HMGCR or FDPS knockout in POP92, POP66, and two additional 3D spheroid models CSC28 and LS174T-S. Two specific sgRNAs were designed to target HMGCR and FDPS, respectively, and both sgRNAs ablated protein expression of their target genes in four colon CSC-enriched spheroid models (Fig. 4A). Depletion of HMGCR or FDPS significantly inhibited CSC-enriched spheroid growth, as determined by cell viability assay (Fig. 4B). Apoptosis assay demonstrated that HMGCR or FDPS knockout both induced a significant increase of early and late apoptotic cells (Fig. 4C). In addition, we found that loss of HMGCR or FDPS significantly impaired sphere formation (Fig. 4D, E) and self-renewal capacity (Fig. 4F) as determined by sphere formation and limiting dilution assays (LDAs). Corroborating our in vitro findings, HMGCR or FDPS deletion significantly inhibited tumor growth in subcutaneous xenograft assay in nude mice (Fig. 4G). To pinpoint the metabolites involved in self-renewal capacity of colon CSCs, we performed rescue assays with downstream metabolites FPP, GGPP, and cholesterol in HMGCR-depleted CSC-enriched spheroids. GGPP and cholesterol, but not FPP, rescued growth inhibitory effect of HMGCR knockout in colon spheroids, implying the requirement of cholesterol and protein prenylation for the growth of CSC-enriched spheroids in vitro (Fig. 4H, I).

**Fig. 5:**
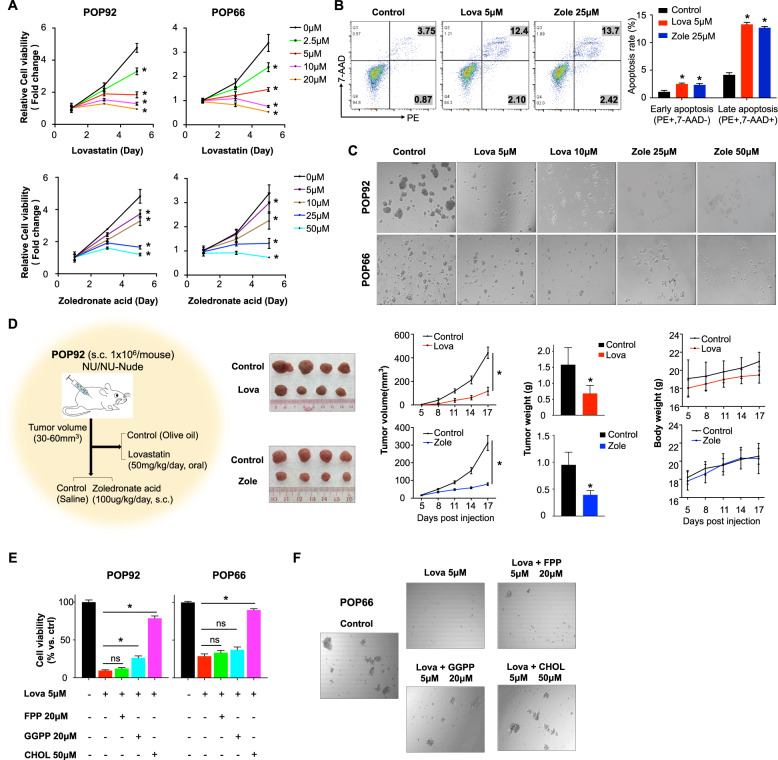
Pharmacological inhibition of cholesterol biosynthesis impairs survival and tumorigenic ability of colon CSC-enriched spheroids. **A** Cell viability, **B** cell apoptosis, and **C** sphere-formation assays of colon CSC-enriched spheroids treated with lovastatin (Lova) or zoledronate acid (Zole) at indicated concentrations as compared to control group. **D** Representative images of excised tumors in control group, 50-mg/kg/day lovastatin or 100-μg/kg/day zoledronate acid group. Body weight, tumor volume and tumor weight were recorded. **E** Effect of cholesterol biosynthesis pathway metabolites on cell viability of colon CSC-enriched spheroids after lovastatin treatment. **F** Representative images of POP66 spheres treated as indicated. Error bar, mean ± SD. For **D**, error bar, mean ± SEM. ns not significant, *P < 0.05 (*t*-test for **A**, **B**, **D**, one-way ANOVA for **E**).

Given the important role of HMGCR and FDPS in the maintenance of colon CSC-enriched spheroids, we next determined if pharmacological targeting of these two genes could suppress their survival. As expected, both lovastatin (HMGCR inhibitor) and zoledronate acid (FDPS inhibitor) significantly inhibited colon spheroid growth in a time- and dose-dependent manner (Fig. 5A). Lovastatin or zoledronate acid also induced cell apoptosis (Fig. 5B), and impaired sphere-formation of colon CSC-enriched spheroids in vitro (Fig. 5C). In addition, both lovastatin and zoledronate acid exerted antitumor growth effect in vivo, with little effect on body weight of mice (Fig. 5D). Consistently, either HMGCR or FDPS depletion reduced intracellular cholesterol levels in POP92 and POP66 spheroids, and POP92-derived xenografts in vivo (Supplementary Fig. 4A, B). Supplementation with cholesterol or GGPP rescued impaired growth of colon CSC-enriched spheroids treated with lovastatin (Fig. 5E, F), confirming the role of cholesterol biosynthesis pathway-derived metabolites in the survival of CSC-enriched spheroids. NCM460, a normal colon epithelial cell line, was less sensitive to lovastatin or zoledronate acid treatment (Supplementary Fig. 5). And lovastatin or zoledronate acid treatment had no significant effect on mice body weight, indicating a potential therapeutic window. Collectively, these results demonstrate the role of cholesterol biosynthesis pathway in contributing to metabolites required for survival and pluripotency of colon CSC-enriched spheroids.

### Cholesterol biosynthesis pathway inhibition deregulates metabolic- and TGF-β-associated signaling cascades in colon CSC-enriched spheroids

**Fig. 6:**
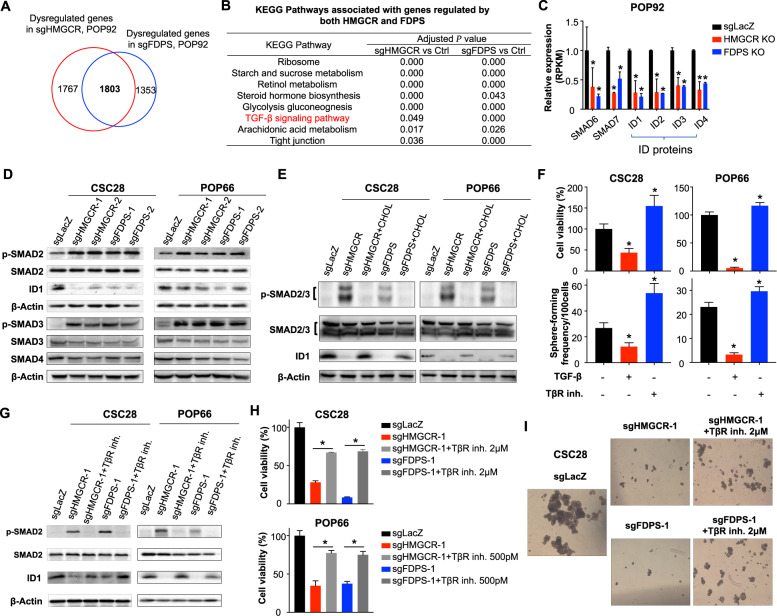
Cholesterol biosynthesis inhibition activates TGF-β signaling to regulate self-renewal of colon CSC-enriched spheroids. **A** Venn diagram showing commonly dysregulated genes in HMGCR- and FDPS-depleted colon CSC-enriched spheroids, based on RNA-seq analysis in POP92. **B** Pathways commonly dysregulated by both HMGCR and FDPS in colon CSC-enriched spheroids. **C** Downregulated TGF-β signaling targets after HMGCR or FDPS depletion in POP92. **D** Western blot analysis of p-SMAD2 (Ser465/467), p-SMAD3 (Ser423/425), SMAD4, and downstream target ID1 in CSC28 and POP66 after depletion of HMGCR or FDPS. **E** Effect of downstream metabolite cholesterol on p-SMAD2/3 and ID1 protein in HMGCR- or FDPS-depleted CSC28 and POP66. **F** Colon CSC-enriched spheroids were treated with TGF-β1 (100 pM for CSC28 and 50 pM for POP66) or TβR inhibitor (LY2109761, 2 μM for CSC28; 500 pM for POP66) for 7 days and the percentage of sphere-forming cells and total cell number were evaluated. **G** Effect of TβR inhibitor on p-SMAD2 and ID1 protein in HMGCR- or FDPS-depleted CSC28. **H** Rescue assays using TβR inhibitor in HMGCR- or FDPS-depleted colon CSC-enriched spheroids. The total cell number were determined by cell viability assays. **I** Representative images of CSC28 spheres treated as indicated. Error bar, mean ± SD. *P < 0.05 (*t*-test for **C**, **E**, one-way ANOVA for **H**).

To probe the molecular basis underlying cholesterol biosynthesis pathway in colon cancer stemness maintenance, we performed RNA-seq analysis to interrogate gene expression profiles in POP92 spheroids after the depletion of HMGCR or FDPS. We identified 3570 and 3156 differentially expressed genes in HMGCR- and FDPS-knockout spheroids, respectively, with 1803 genes commonly deregulated under both conditions (adjusted *P* < 0.05 and fold change >2) (Fig. 6A). Gene set enrichment analysis (GSEA) revealed that HMGCR or FDPS knockout commonly downregulated metabolic pathways including glycolysis, gluconeogenesis, starch and sucrose metabolism, retinol metabolism, and arachidonic acid metabolism, together with TGF-β signaling pathway (Fig. 6B and Supplementary Fig. 6A, B). Analysis of RNA-seq dataset showed that HMGCR or FDPS knockout led to significant downregulation of inhibitory SMADs (SMAD6 and SMAD7) and their downstream targets ID proteins, that are part of TGF-β signaling cascade (Fig. 6C and Supplementary Fig. 6C). ID proteins are key regulators of CSCs in a number of cancer types [11, 26, 27], suggesting that cholesterol biosynthesis may regulate colon CSC self-renewal via TGF-β-ID signaling. We thus investigated TGF-β signaling upon HMCGR or FDPS knockout. Western blot revealed the upregulation of phosphorylated SMAD2 (p-SMAD2), phosphorylated SMAD3 (p-SMAD3) in HMGCR or FDPS-knockout POP66 and CSC28, indicating the activation of TGF-β signaling (Fig. 6D). Concomitantly, ID1 protein abundance was reduced upon HMGCR or FDPS depletion (Fig. 6D), consistent with a repressive role of TGF-β signaling on ID proteins [28, 29]. In line with these findings, lovastatin or zoledronate acid treatment in POP92 upregulated p-SMAD2 expression, whilst downregulating SMAD6/7 and ID proteins (Supplementary Fig. 6D, E). Cholesterol supplementation reversed the effect of HMGCR or FDPS depletion on p-SMAD2/3 and ID1 (Fig. 6E), implying that cholesterol biosynthesis pathway regulates TGF-β-ID axis via its end point metabolite. TGF-β is a potent epithelial–mesenchymal transition (EMT) inducer. Consistently, E-cadherin was downregulated by HMGCR or FDPS knockout, in line with increased EMT (Supplementary Fig. 6F). No significant change was observed for Snail and Zeb1, implying that sgHMGCR or sgFDPS may partially repress epithelial characteristics in CSC-enriched spheroids.

To determine whether modulation of TGF-β signaling by cholesterol biosynthesis is important for colon CSC-enriched spheroids, we evaluated the effect of TGF-β on the self-renewal capacity. CSC28 and POP66 were treated with TGF-β1 or a TβR inhibitor to induce or suppress TGF-β signaling, respectively. TGF-β1 attenuated cell viability and sphere formation in both models, while TβR inhibitor exerted an opposite effect (Fig. 6F). Moreover, the blockade of HMGCR or FDPS-knockout-induced TGF-β signaling by TβR inhibitor (Fig. 6G) rescued cell viability and sphere-forming capacity (Fig. 6H, I) of CSC28 and POP66, indicating that TGF-β signaling functions downstream of cholesterol biosynthesis pathway to modulate CSC self-renewal. Overall, our data indicate that cholesterol biosynthesis pathway represses TGF-β signaling to induce self-renewal capacity of colon CSC-enriched spheroids.

### Cholesterol biosynthesis inhibitors synergize with chemotherapeutics to suppress colon cancer growth

**Fig. 7:**
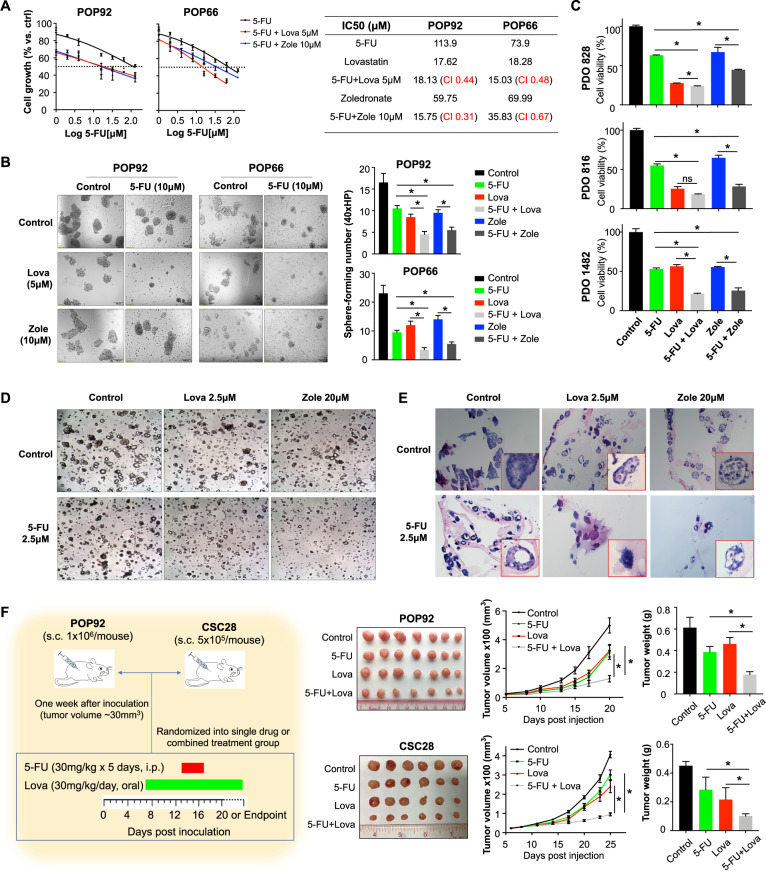
Cholesterol biosynthesis inhibitors synergize with 5-FU to suppress colon cancer in vitro and in vivo. **A** The dose–response curve of indicated drug or drug combination(s) was plotted and half maximal inhibitory concentration (IC_50_) was determined. Combination index (CI) for 5-FU and cholesterol biosynthesis inhibitors (lovastatin and zoledronate acid) were calculated according to Chou–Talalay’s method at 72-h timepoint. CI < 1, CI = 1, and CI > 1 indicate synergistic, additive, and antagonistic effects, respectively. **B** Sphere-formation frequency of colon CSC-enriched spheroids after treatment with 5-FU and lovastatin/zoledronate acid, alone or in combination. **C** Cell viability of colon cancer PDOs after treatment with 5-FU and lovastatin/zoledronate acid, alone or in combination. **D** Microscopic images and **E** H&E staining of colon cancer PDO 828 treated with 5-FU and lovastatin/zoledronate acid, alone or in combination at indicated concentrations for 6 days. **F** In vivo xenograft assay in nude mice bearing colon CSC-enriched spheroids-derived tumors. Mice were treated with vehicle, 5-FU, lovastatin, or 5-FU plus lovastatin at low dosages as indicated. Representative images, tumor volume, and tumor weight were shown. Error bar, mean ± SD. For **F**, error bar, mean ± SEM. *P < 0.05 (one-way ANOVA).

Cancer stemness are key drivers of therapeutic resistance in various cancers, including colon cancer [4, 30]. Concordantly, we found that POP92 and POP66 spheroids were considerably more resistant to conventional chemotherapeutic drugs, such as 5-FU and oxaliplatin, as compared to HCT116, HT29, and SW480 cell lines (Supplementary Fig. 7A, B). Given that cholesterol biosynthesis inhibitors effectively suppressed POP92 and POP66 spheroid growth and self-renewal as shown in Fig. 5A–F, we thus hypothesized that lovastatin and zoledronate acid treatment could sensitize them to conventional therapy. We therefore treated POP92 and POP66 spheroids with lovastatin or zoledronate acid alone or in combination with 5-FU, a commonly employed chemotherapeutic drug in colon cancer (Fig. 7A). Both lovastatin and zoledronate acid co-operatively inhibited POP92 and POP66 viability in conjunction with 5-FU, as evidenced by combination indices of <1 for all drug combinations (Fig. 7A). The co-treatment of lovastatin or zoledronate acid with 5-FU significantly reduced the sphere-formation capacity of POP92 and POP66 spheroids compared to single drug treatment (Fig. 7B), implying synergism between cholesterol biosynthesis inhibition and 5-FU. To determine whether cholesterol biosynthesis inhibitors confer a therapeutic benefit in combination with 5-FU in colon cancer, we determined the effect of the aforementioned drug combination(s) in three colon cancer patient-derived organoids (PDOs). Consistently, cholesterol biosynthesis inhibitors enhanced the anticancer effect of 5-FU in all the PDOs (Fig. 7C). Microscopic images and H&E staining showed decreased organoid-forming cells and impaired organoid structure in PDO 828 after treatment with 5-FU plus lovastatin or zoledronate acid (Fig. 7D, E). To corroborate our in vitro data, we determined the efficacy of 5-FU, lovastatin or their combination in low dosage using POP92-derived xenografts in nude mice. As shown in Fig. 7F, 5-FU or lovastatin treatment alone slightly inhibited tumor growth, whereas their combination significantly suppressed xenograft growth compared to other groups. Collectively, these data indicate targeting cholesterol biosynthesis pathway sensitizes CSC-enriched spheroids to chemotherapy.

Finally, we determined whether targeting cholesterol biosynthesis pathway is a viable approach in drug-resistant cells. To this end, we evaluated the growth inhibitory effect of cholesterol biosynthesis inhibitors in two drug-resistant cell lines, HCT15/FU and SW620/FU, which were resistant to 5-FU. Cell viability assays showed that lovastatin or zoledronate both significantly impaired cell viability of HCT15/FU and SW620/FU (Supplementary Fig. 8). Hence, cholesterol biosynthesis represents a druggable target for colon cancer cells exhibiting stemness or drug resistance characteristics.

## Discussion

Cancer stemness are considered the basis of drug resistance, tumor relapse, and metastasis. Hence, it is critical to unravel novel molecular targets to eradicate cells with stemness characteristics. Here, we utilized a CRISPR/Cas9 Epi-Drug library to systematically screen genetic vulnerabilities in colon CSC-enriched spheroid models and identified that cholesterol biosynthesis pathway is essential for spheroid survival. We validated functional role of cholesterol biosynthetic genes HMGCR and FDPS for colon CSC-enriched spheroid viability and self-renewal; and established druggability of the two targets with FDA-approved drugs. Finally, we demonstrated that combining cholesterol biosynthesis inhibitor with conventional chemotherapy was synergistic in suppressing CSC-enriched spheroids and overcomes drug resistance in colon cancer cells, implying that cholesterol biosynthesis is a druggable pathway for the chemosensitization of colon cancer.

Our targeted CRISPR screens identified cholesterol biosynthesis as essential for survival and pluripotency in colon cancer patient-derived CSC-enriched spheroids. Concordantly, we found the upregulation of cholesterol biosynthesis genes in CSC-enriched spheroids and primary colon tumors. Genetic ablation or pharmacological inhibition of two critical molecular targets, HMGCR and FDPS, confirmed functional importance of this pathway in colon CSC-enriched spheroids. Cholesterol biosynthesis pathway, also known as the mevalonate pathway, is a multistep process that produces cholesterol and other intermediates such as GGPP, FPP, and sterols. We found that supplementation with cholesterol or GGPP rescued growth and pluripotency of colon CSC-enriched spheroids with knockout or blockade of HMGCR, thus substantiating that cholesterol biosynthesis-derived intermediate metabolites are directly involved in the maintenance of colon cancer stemness.

Our results indicate that cholesterol biosynthesis-mediated cancer stemness might involve two distinct mechanisms. The first one is directly associated with cholesterol, while an alternative mechanism involves GGPP-dependent protein prenylation. In agreement with our findings, multiple lines of evidence suggest the role of cholesterol in intestinal CSCs regulation. For example, cholesterol was found to promote intestinal stem cell growth and tumorigenesis in intestinal-specific Srebf2 transgenic mice; and biosynthesis of cholesterol played a determinant role for tumor formation in *Apc*^*min/+*^ mice model [31]. Apart from intestinal CSCs, cholesterol biosynthesis was shown to enhance self-renewal and tumorigenic capacity of mammosphere and neurosphere [32–34], implying an important role of cholesterol in pluripotency in multiple malignancies. GGPP, on the other hand, serves as a substrate for prenylation of small GTPases, such as Ras, Rho, and Rac [25], as a prerequisite step necessary for membrane localization and activation. GTPases activation also endows cancer stemness properties, as reported by others [32]. Taken together, cholesterol biosynthesis pathway is activated in colon CSC-enriched spheroids to sustain pluripotency, with cholesterol and GGPP as the major effectors.

The underlying mechanism linking cholesterol biosynthesis pathway to colon CSC-enriched spheroids survival is unclear. We identified TGF-β signaling as a mechanistic link between upregulated cholesterol biosynthesis and colon CSC-enriched spheroids survival by RNA sequencing analysis. Cholesterol deprivation by genetic inhibition or pharmacological blockade of HMGCR or FDPS activated TGF-β signaling to repress the expression of ID proteins, which has an established role in driving stemness [11, 35, 36]. Consistently, TGF-β impaired self-renewal capacity, while the inhibition of TβR promoted spheroid growth and rescued the inhibitory effect of cholesterol deprivation. Besides, BMPs as part of TGF-β superfamily, might be also involved in this process as statins were reported to activate BMP2 expression to induce colon cancer cell apoptosis [37].

Cholesterol-derived metabolites such as oxysteroids or steroid hormones have also been reported to modulate oncogenic signaling pathways such as Hedgehog, Wnt, or MAPK [38]. However, in the context of colon CSC-enriched spheroids, cholesterol appears to be the active metabolite, as direct cholesterol addition overcome the functional effects of HMGCR/FDPS knockout. In agreement with our data, cholesterol itself has been shown to promote intestinal stem cell proliferation and tumorigenesis in *Apc*^*min*^ mice [31]. Several studies have implied a potential role of cholesterol on the regulation of TGF-β signaling. For instance, cholesterol could induce the rapid degradation of TβRs localized in lipid rafts and repress TGF-β responsiveness. Conversely, the inhibition of cholesterol biosynthesis pathway markedly increased the expression of TβRs, TGF-β1, and TGF-β in different cell types [39–42]. Disruption of cholesterol biosynthesis by Nsdhl knockout or statins treatment also induced SREBP1-dependent Tgfb1 expression and autocrine TGF-β-SMAD2/3 signaling in pancreatic cancer cells [43]. Cholesterol thus negatively modulates TGF-β signaling via diverse mechanisms, which in turn, can promote the survival of CSC-enriched spheroids.

Cholesterol biosynthesis pathway represents an attractive therapeutic target, as it has well-defined molecular targets and FDA-approved drugs that enables potential drug repurposing. HMGCR is a primary target of statins, a large class of cholesterol-lowering drugs commonly prescribed to individuals with hyper-cholesterolemia, whereas FDPS is targeted by bisphosphonates, a class of drugs used for treating bone diseases [44]. Here, we demonstrated that therapeutic targeting of HMGCR or FDPS with lovastatin or zoledronate acid effectively suppressed colon cancer spheroids self-renewal in vitro and tumorigenic potential in vivo. Others have also proposed statins or bisphosphonates as potential antineoplastic agents in a variety of cancers [45–47]. Nevertheless, we systematically identify and formulate the inhibitory effect of these drugs on colon CSC-enriched spheroids. Importantly, targeting of cholesterol biosynthesis appeared safe for mice in our study. In agreement with our observations, others showed that cholesterol biosynthesis inhibition did not impair normal intestinal crypt proliferation [31], implying a therapeutic window for selective targeting of colon cancer. Indeed, epidemiological studies and meta-analyses of clinical trials have shown that statins are chemopreventive and protective against colon cancer [48–50]. Our results thus advocate for the strategy of utilizing statins to target colon cancer cells with stemness traits.

Numerous studies have reported that traditional chemotherapy preferentially targets fast-proliferating differentiated cancer cells, whilst sparing CSCs [13]. Concordantly, we demonstrated that colon CSC-enriched spheroids were highly resistant to 5-FU and oxaliplatin compared to colon cancer cell lines. Combinatorial CSC-targeting therapy and conventional chemotherapy represent a promising strategy for treating cancer [6], as both CSCs and non-CSCs are simultaneously targeted. We found a synergistic anticancer effect between cholesterol biosynthesis inhibitors (lovastatin/zoledronate acid) and chemotherapy in colon CSC-enriched spheroids, primary colon tumor organoids, and spheroid-derived xenografts, all of which models the heterogenous nature of CSCs and non-CSCs population in human tumors. Such synergism is likely the consequence of impaired self-renewal and augmented cell differentiation by cholesterol biosynthesis inhibitors. Such phenomenon was also reported in acute myelocytic leukemia (AML). Cholesterol levels are abnormally induced in AML cells after exposure to chemotherapy in vitro and blocking this cholesterol response was further shown to sensitize AML cells to standard therapeutic drugs [51]. Collectively, our work suggests a novel therapeutic approach for colon cancer treatment.

In conclusion, our work highlighted a crucial role of cholesterol biosynthesis pathway in the maintenance of colon CSC-enriched spheroids and established this pathway as an attractive therapeutic target for the eradication of colon cancer cells with stemness and drug resistance traits. The combination of cholesterol biosynthesis inhibitors plus conventional chemotherapy holds promise for colon cancer treatment.

## Materials and methods

### Cell culture and reagents

Human colon CSC-enriched spheroid models (POP92, POP66, CSC28, LS174T-S) and colon cancer PDOs (PDO 828, 1482, 816) were kindly provided by Dr CAOB in Princess Margaret Cancer Center, University of Toronto. POP92, POP66, and CSC28 were derived from a primary colon tumor specimen and two liver metastases of colon adenocarcinoma, respectively, and cultured in 3D suspension state in previously established serum-free, growth factor-enriched medium that enriches for CSCs [7]. LS174T-S was enriched from a commercial colon cancer cell line LS174T and cultured in aforementioned medium. PDOs were derived from a primary tumor (PDO 828) and two lung metastases (PDO1482, 816) of colon cancer and cultured in 3D serum-reduced matrigel (Corning, NY, USA) in advanced DMEM/F12 medium (Gibco, CA, USA) supplemented with 1% penicillin-streptomycin (Thermo Fisher, MA, USA), 1x HEPES (Gibco), 1x GlutaMAX-1 (Gibco), 1x B-27 (Gibco), 1.25-mM N-Acety l-L-dysteine (Sigma-Aldrich, MO, USA), 10-nM [Leu15]-Gastrin I (Sigma-Aldrich), 50-ng/ml mEGF (Gibco), 100-ng/ml mNoggin (Peprotech, NJ, USA), and 0.5-µM A83-01 (Tocris Bioscience, MN, USA). HCT15/FU and SW620/FU are two laboratory-developed drug-resistant colon cancer cell lines that could survive under high concentrations of 5-FU, and they were purchased from iCell Bioscience Incorporated Company (Shanghai, China). Colon cancer cell lines (HCT116, HT29, SW480) and HEK-293FT were purchased from ATCC (MD, USA) and cultured according to protocols. All the cell lines were authenticated by STR profiling and tested for mycoplasma contamination regularly. Chemicals and reagents see also Supplementary Materials and Methods. PCR primers and sgRNAs are listed in Supplementary Tables S4 and S5.

### CRISPR dropout screens

Pooled Epi-Drug sgRNA library was designed and cloned into lentiGuide-puro plasmid by He lab. Colon CSC-enriched spheroids were transduced with a predetermined volume of the pooled sgRNA lentiviral supernatant. About 1 day after transduction, the cells were selected with puromycin for 2 days and further cultured in stem cell primitive medium without puromycin. Approximately 15 million cells were harvested for DNA extraction, next-generation sequencing, and data analysis on “Day 0,” “Day 8,” and “Day 16.” See also Supplementary Materials and Methods.

### Sphere-formation assays and in vitro LDAs

Colon CSC-enriched spheroids were digested into single cell suspension, counted, and seeded in 6-well plates at 3000 cells per well in suspension state. Sphere number and diameter were evaluated after 5–7 days of culture. For in vitro LDAs, viable spheroid cells were sorted into 96-well plates at densities of 10, 5, or 1 cell per well, using FACS cell sorters, followed by drug treatments. About 2 weeks later, wells containing spheres were counted and the results were analyzed using a web-based tool (ELDA, http://bioinf.wehi.edu.au/software/elda/index.html).

### Xenograft assays

The xenograft animal experiments were carried out under the approval of Animal Experimentation Ethics Committee in the Chinese University of Hong Kong (Ref no. 19-098-MIS). Colon CSC-enriched spheroids (1 × 10^6^ cells/tumor for POP92 and LS174T-S, 5 × 10^5^ cells for CSC28) were subcutaneously injected into right flanks of 4–6-week-old male nude mice. To investigate the tumorigenesis ability after cholesterol biosynthesis genes depletion, cells with/without HMGCR/FDPS were injected subcutaneously (*n* = 6 or 7), and tumors were measured and recorded every 2–3 days until the end point. For drug treatment assays, tumor-bearing mice were randomized into control group and treatment group when the average tumor volume reached 30–60 mm^3^ (4–8 mice per group). To test the anticancer effect of single drug treatment, lovastatin (50 mg/kg per day, oral gavage), or zoledronate acid (100 µg/kg per day, subcutaneous) was given to mice in treatment groups (*n* = 4). To investigate combinatorial effect of 5-FU and lovastatin, four groups (*n* = 7) were included: control (olive oil per day + saline, 5 times), 5-FU (30 mg/k/day, 5 times, intraperitoneal, i.p.), lovastatin (30 mg/kg/day) or their combination (5-FU 30 mg/kg/day, 5 times, i.p. and lovastatin 30 mg/kg/day). Body weight and tumor volume were measured every 2–3 days until the end point.

### RNA sequencing data analysis

Total RNA from POP92 cells were sequenced on Hiseq2500 (Novogene, Beijing, China) after the indicated treatments. Reads were aligned with HISAT2 v2.1.0 to hg38 human reference genome, and raw count of each sample was evaluated by StringTie v0.11.2. R DESEq2 package was employed for the normalization and transformation of gene expression. GSEA was also performed using gene sets from KEGG pathways in the Molecular Signatures Database [52].

### Statistics

All experiments were done at least twice independently. Statistical analyses were justified as appropriate in terms of assumptions of tests and variation estimate, performed on the software Prism 7 (GraphPad). Two-tailed unpaired *t*-tests or paired *t*-tests were performed for comparisons of two groups. One-way ANOVA (for more than 2 group comparisons) was performed followed by the Bonferroni multiple comparison post test. For all comparisons, a *P* value of <0.05 was considered statistically significant. Data are presented as mean ± SD (ns not significant, *P < 0.05).

## Supplementary information

Supplementary Information
