# Supplementary material for: CRISPR screens identify cholesterol biosynthesis as a therapeutic target on stemness and drug resistance of colon cancer
Source: Oncogene. 2021 Oct 7;40(48):6601–13. doi: 10.1038/s41388-021-01882-7 (PMC8639446; doi:10.1038/s41388-021-01882-7)
Supplement: Supplementary file 1 — Supplementary Information [file 41388_2021_1882_MOESM1_ESM.pdf]

Supplemental Information to

CRISPR screens identify cholesterol biosynthesis as a therapeutic target on stemness and drug resistance of colon cancer

Shanshan Gao[1,2], Fraser Soares[2], Shiyan Wang[2], Chi Chun Wong[1], Huarong Chen[1], Zhenjie Yang[1], Weixin Liu[1], Minnie YY Go[1], Musaddeque Ahmed[2], Yong Zeng[2], Catherine Adell O'Brien[2], Joseph JY Sung[1], Housheng Hansen He[2,3*], Jun Yu[1*]

Supplementary Materials and Methods

Supplementary Figure S1-8

Supplementary Table S1-5

Supplementary Materials and Methods

Chemicals and Reagents

5-FU (F8423), oxaliplatin (O9512), zoledronate acid monohydrate (SML0223), geranylgeranyl pyrophosphate ammonium salt (G6025), farnesyl pyrophosphate ammonium salt (F6892) and cholesterol-water soluble (C4951) were all from Sigma-Aldrich. Lovastatin was obtained from J&K scientific (Beijing, China). Recombinant human TGF-beta 1 protein (240-B-002/CF), TGF-β receptor (TβR) I/II kinase inhibitor LY2109761 (A11133) were separately purchased from R&D systems (MN, USA) and Adooq Bioscience (CA, USA). All the compounds were dissolved according to the manufacturer's protocols. PCR primers and sgRNAs were synthesized by Thermo Fisher and their sequences are listed in **Supplementary Table S4 and S5.**

CRISPR Screening

sgRNA library design, synthesis and amplification. Pooled Epi-Drug sgRNA library was designed using CRISPR-DO tool by He lab and synthesized as 73-mer oligonucleotides (CustomArray, NJ, USA), amplified by PCR and then cloned into lentiGuide-puro plasmid (a gift form Feng Zhang, Addgene 52963). During transformation, over 300-fold coverage were maintained (>4 million colonies) to achieve an adequate library representation. The plasmid was sequenced to confirm the representation of each sgRNA.

sgRNA library viral packaging. 293FT cells ($1x10^7$) were seeded in each 15cm dish

with 70-90% confluency. Lentivirus particles were generated with library plasmids, psPAX2 and pMDG.2 at a ratio of 20:15:6μg using X-tremeGENETM HP transfection reagent (Sigma-Aldrich). Viral supernatants were harvested at 48h and 72h, and then concentrated at a ratio of 10:1 using PEG-it virus precipitation solution (System Biosciences, CA, USA).

CRISPR dropout screens. Cas9 lentiviral particles were first transduced into colon CSC-enriched spheroids. After blasticidin selection (10 μg/ml) for 2 weeks, Cas9-expressing spheroids were used for library transduction. To validate the editing efficiency of the CRISPR/Cas9 system, two specific sgRNAs targeting METTL3 were used to assess knockout efficiency. For library transduction, Cas9-expressing CSCs were seeded in 6-well plates ($3x10^6$ cells per well). The concentrated viruses were added to the cell suspension with 8 μg/ml polybrene (Sigma). The plates were centrifuged at 37°C, 1000 rpm for 1-2 h, and then incubated at 37°C incubator for 8h. Transduced cells were then resuspended in 40 ml stem cell primitive medium in T175 flask. At 24h post-transduction, puromycin was added into the medium at 3 μg/ml for POP92 and 4 μg/ml for POP66. At 72h post-transduction, selective medium was replaced by medium without puromycin and 15 million cells were harvested for DNA extraction and data analysis as “Day 0” timepoint. 8 days and 16 days later, cells (about 30 million) were collected for DNA extraction separately as “Day 8” and “Day 16” timepoint. Purified DNA were analyzed by next generation sequencing (NGS).

NGS data analysis. NGS data was first aligned to library sgRNA sequences with bowtie version 1.2.2 (http://bowtie-bio.sourceforge.net/index.shtml). Read count for every sgRNA was computed by a custom python script. A web-based tool MAGeCK was used to estimate significantly depleted or enriched genes in each timepoint. Gene set enrichment analysis was done by a web-based gene set analysis toolkit (WebGestalt, http://www.webgestalt.org/). Gene sets were analyzed using overrepresentation enrichment analysis (ORA), based on the functional Reactome database.

Cell viability assays and cell apoptosis assays

Cells were treated with indicated compounds in 96-well plate. Cell viability was measured with the CellTiter one solution cell proliferation assay (Promega) for spheres, and for PDOs, CellTiter-Blue cell viability assay (Promega, WI, USA) was adopted. Cell apoptosis were estimated by flow cytometry using Annexin-PE/ 7-aminoactinomycin D (7-AAD) staining kit (BD Biosciences, CA, USA).

Western blot and immunohistochemistry (IHC) analysis

Colon tumor specimens and matched non-tumor tissues were kindly provided by Professor NG Siu Man, Simon, the Prince of Wales Hospital, Hong Kong, with prior ethic approval and informed consent. Protein extracts (30 μg) were subjected to SDS-PAGE and then transferred to nitrocellulose membrane for western blot analysis. Multiple tumor tissue pairs were embedded in parafilm, sectioned and analyzed by IHC staining. Immunostaining intensity was determined using Allred IHC score system for

intensity score (negative, 0; weak, 1; intermediate 2; strong 3) and proportion score (no cells, 0; <1% of cells, 1; <1/10 of cells, 2; <1/3 of cells, 3; <2/3 of cells, 4; 100% of cells, 5). The following antibodies were used: HMGCR (13533-1-AP, Proteintech, IL, USA), HMGCS1 (194971, Abcam, Cambridge, UK), SELQ (12544-1-AP, Proteintech), FDPS (16129-1-AP, Proteintech), CD133 (16518, Abcam), CD133 (19898, Abcam), EphB2 (AF647, R&D Systems), phospho-SMAD2 (Ser465/467) (#3108, Cell Signaling Technology (CST), MA, USA), SMAD2 (#5339, CST), phospho-SMAD3 (#9520, CST), SMAD3 (#66516-1-Ig, Proteintech), phosphor-SMAD2/3 (#8828, CST), SMAD2/3 (#8685T, CST), SMAD4 (10231-1-AP, Proteintech)， ID1 (18475-1-AP, Proteintech), Zeb1 (#3396T, CST), E-cadherin (#14472, CST), N-cadherin (#4061, CST), Snail (#3879, CST).

Flow cytometry

Colon CSC-enriched spheroids were digested into single cell suspension, washed with 0.1%BSA-PBS, and stained with fluorescent human antibodies to CD133 (Miltenyi Biotec) and CD44 (BD Pharmingen). Propidium iodide (1ug/mL) was used to identify viable cells. Mouse IgG1 conjugated to allophycocyanin or phycoerythrin was used as isotype control. Flow cytometry and analysis were on a Becton Dickinson LSR II.

Total cholesterol measurement

Cholesterol/Cholesteryl ester assay kit (Abcam, ab65359) provides a simple tool for sensitive quantification of total cholesterol (cholesterol and cholesterol esters) or free

cholesterol by colorimetric or fluorometric method. Cells (about $1x10^6$) were harvested after indicated treatments for lipid extracted and then subjected to fluorometric assay (Ex/Em=535/587nm) or colorimetric assay (OD570nm) according to the manufacturer protocol.

Drug synergy analysis with Chou-Talalay combination index

Chou-Talalay method was used for the analysis of drug combination effect. Combination index (CI) score was used to estimate the interaction between two drugs with quantitative definition for synergism (CI<1), additive (CI=1) and antagonism (CI>1) effects.

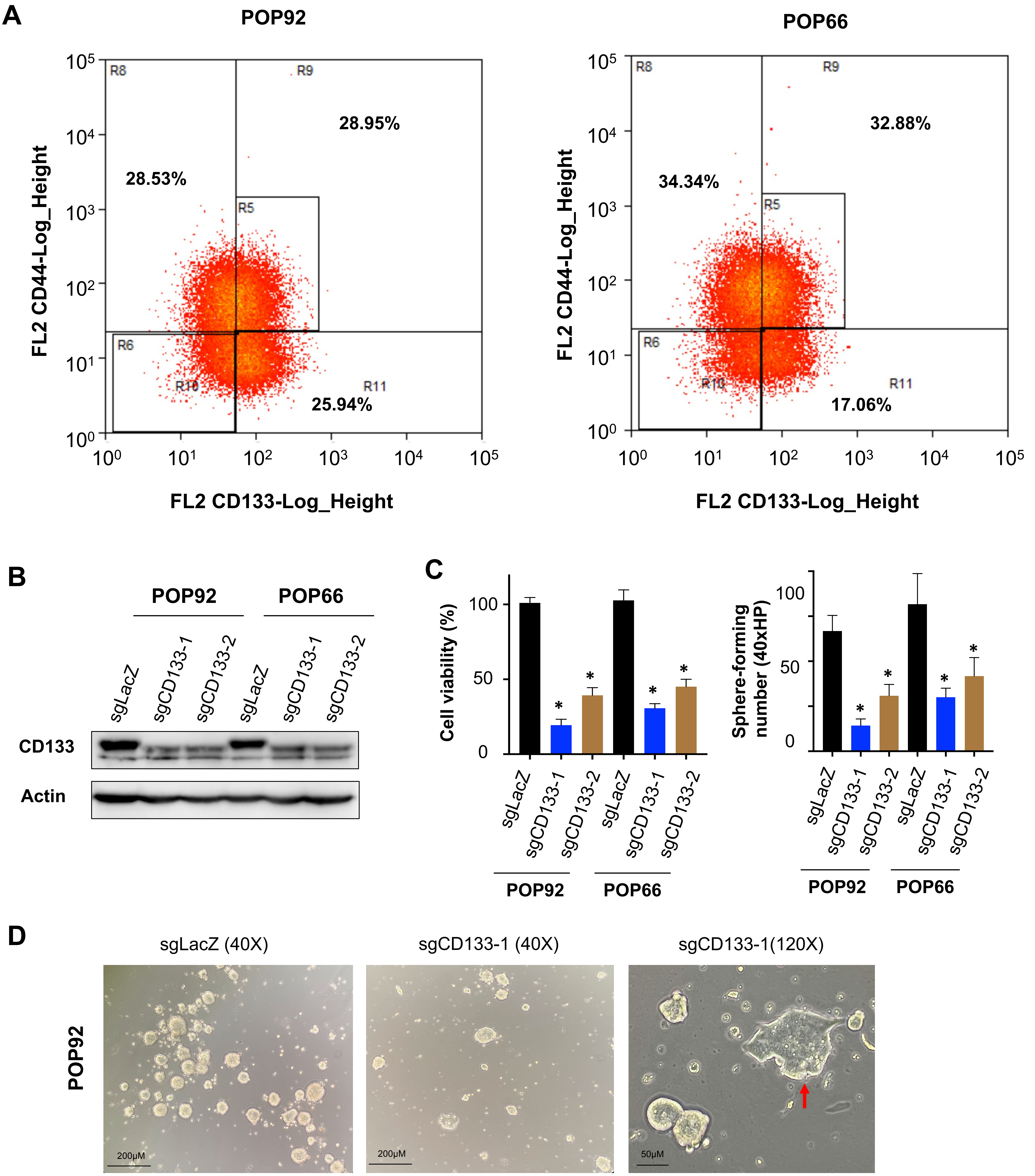

Supplementary Figure 1. Characteristics of stemness markers in colon CSC-enriched spheroid models and the effect of CD133 depletion on cell proliferation and self-renewal ability of colon CSC-enriched spheroids. A, Characterization of stemness markers CD133 and CD44 in colon CSC-enriched spheroid models by flow cytometry. **B,** Colon CSC-enriched spheroids were transduced with sgRNAs targeting CSC marker CD133. Knockout efficacy was evaluated by western blot. **C,** Cell viability and sphere-formation assays of colon CSC-enriched spheroids after depletion of CD133. Representative images under the light microscope were shown in **Panel D**. Red arrow shows cells are attached to the flask and expand around similar as 2D differentiated cells. Error bar, mean ± SD. *P<0.05 (students' t test).

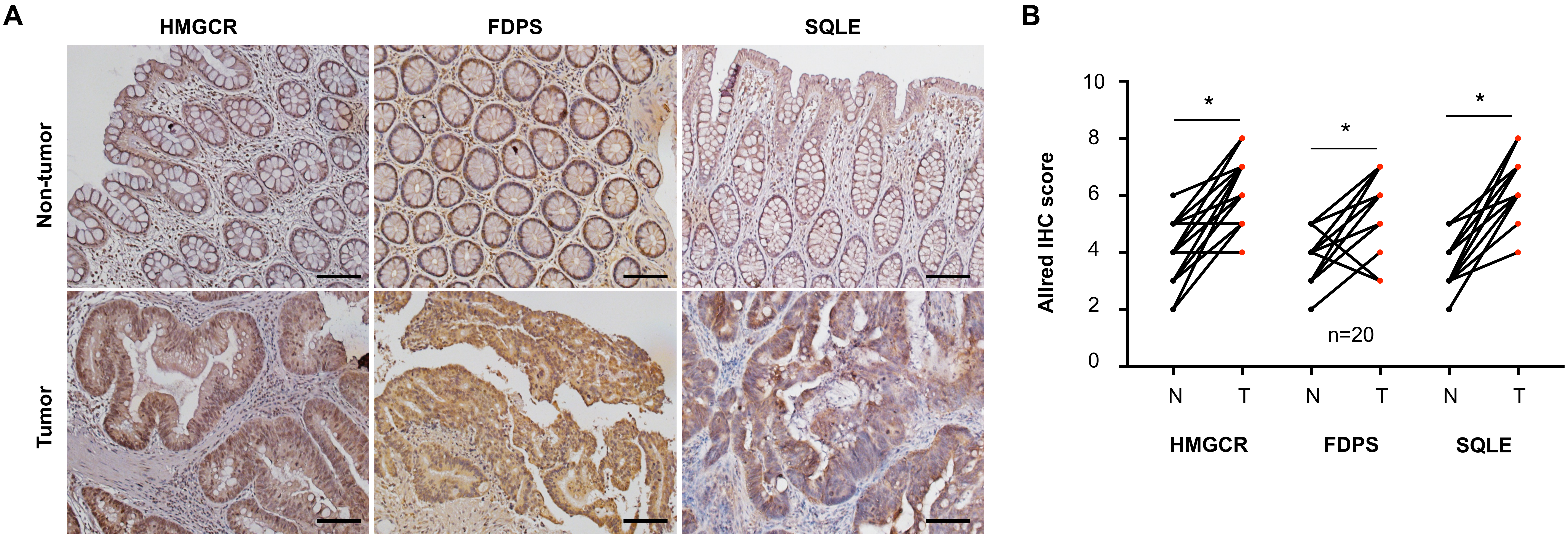

Supplementary Figure 2. IHC analysis of several key cholesterol biosynthetic genes. A, IHC analysis was used to evaluate expression of HMGCR, FDPS and SQLE in 20 pairs of tumor (T) and matched non-tumor (N) tissues. Representative images are shown (scale bar, 100μM). **B,** IHC analysis scores of indicated genes were shown and analyzed by paired t-test. * P<0.05 (paired t-test).

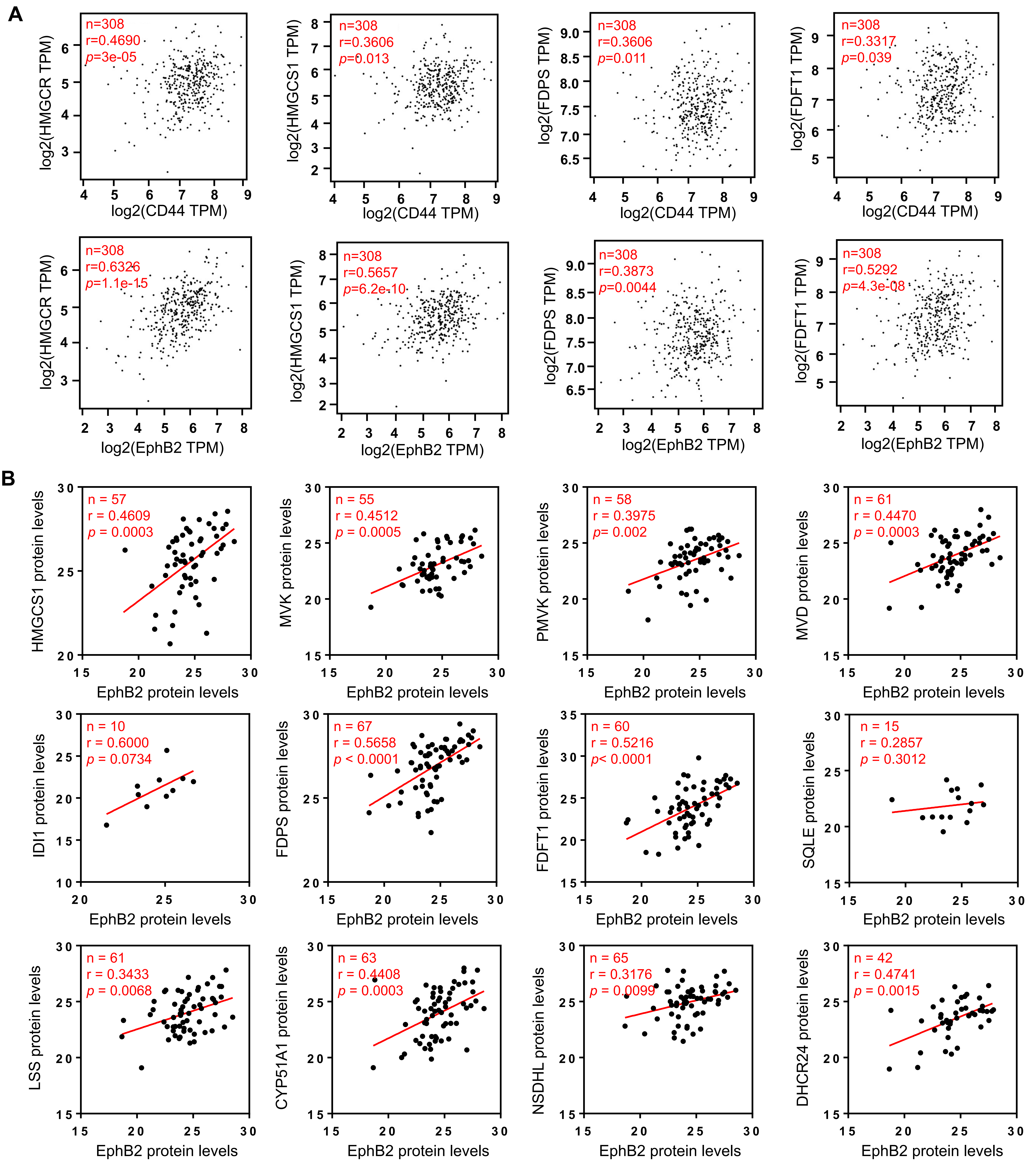

Supplementary Figure 3. Correlation analysis between cholesterol biosynthetic genes and stemness markers CD44 or EphB2. A, Cholesterol biosynthetic genes HMGCR, HMGCS1, FDPS, FDFT1, are positively correlated with stemness marker EphB2 and CD44 at mRNA level in TCGA colorectal cancer (COADREAD, n=308). **B,** Correlation between EphB2 and cholesterol pathway enzymes, HMGCS1, MVK, PMVK, MVD, IDI1, FDPS, FDFT1, SQLE, LSS, CYP51A1, NSDHL, DHCR24, were analyzed at protein level (mass spectrometry by CPTAC) in colon cancer specimens obtained from TCGA RPPA database (n=indicated number).

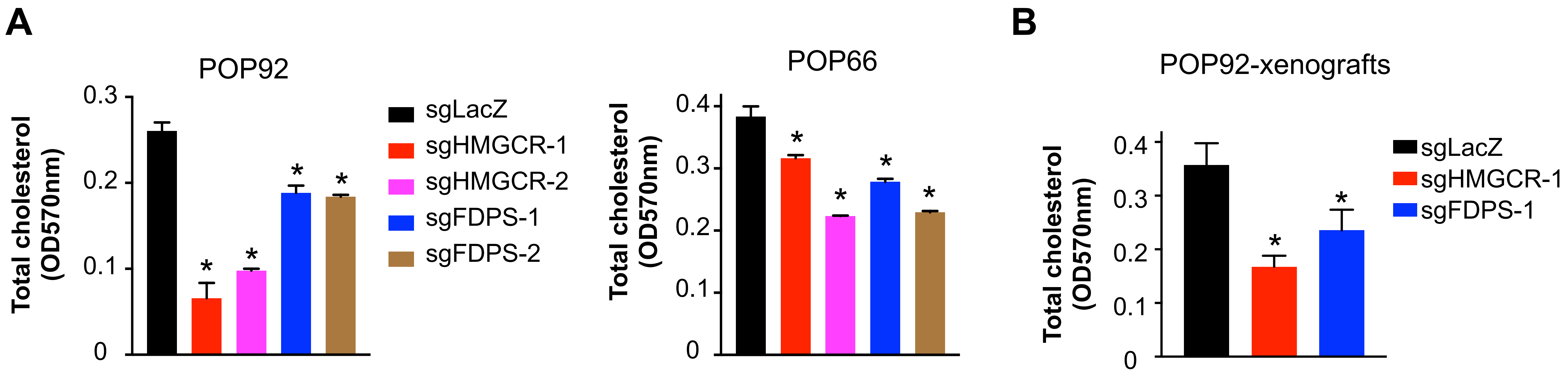

Supplementary Figure 4. The effect of HMGCR/FDPS depletion on total cholesterol level *in vitro* and *in vivo*. A-B, Total cholesterol levels were measured after HMGCR/FDPS knockout in colon CSC-enriched spheroids and POP92-derived xenografts, using cholesterol/cholesteryl ester assay kit. *P < 0.05. Error bar, mean ± SD.

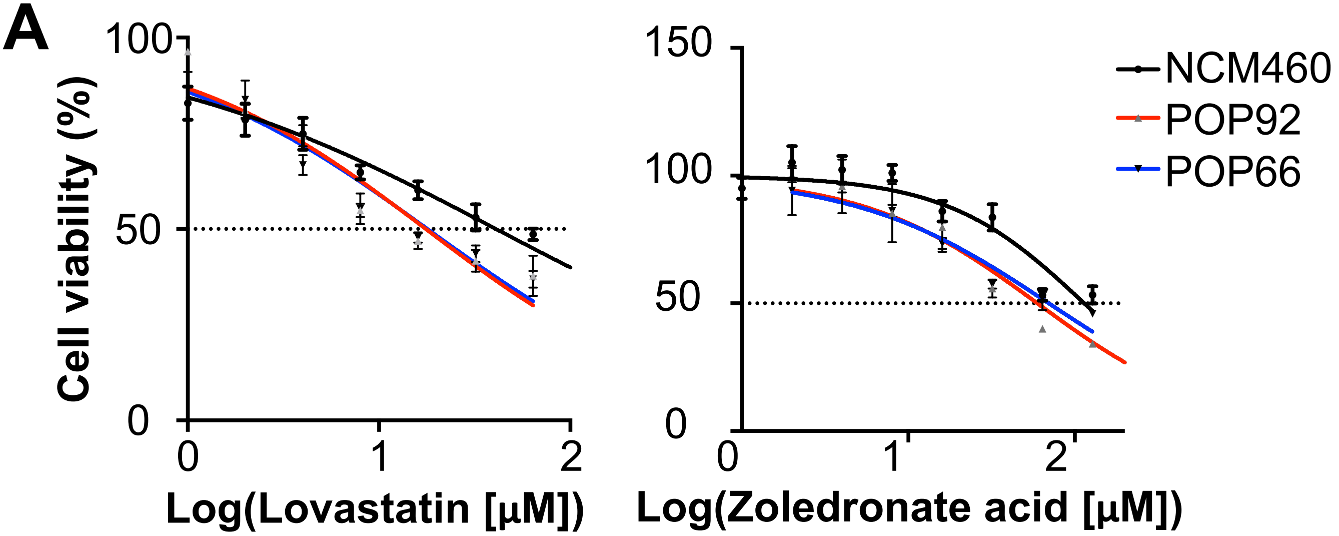

IC50 (µM)	POP92	POP66	NCM460
**Lovastatin**	17.62	18.28	40.95
**Zoledronate acid**	59.75	69.99	114.2

Supplementary Figure 5. The effect of HMGCR/FDPS blockade in colon CSC-enriched spheroids and normal epithelial cell line. A, lovastatin and zoledronate acid dose response curves and IC50 value for colon CSC-enriched spheroids and NCM460. These cells were exposed to a serial concentration of lovastatin or zoledronate acid for 72hours, and cell viability was measured using MTS-based proliferation assay. All values (upper panels) are normalized to untreated control. Error bar, mean ± SD.

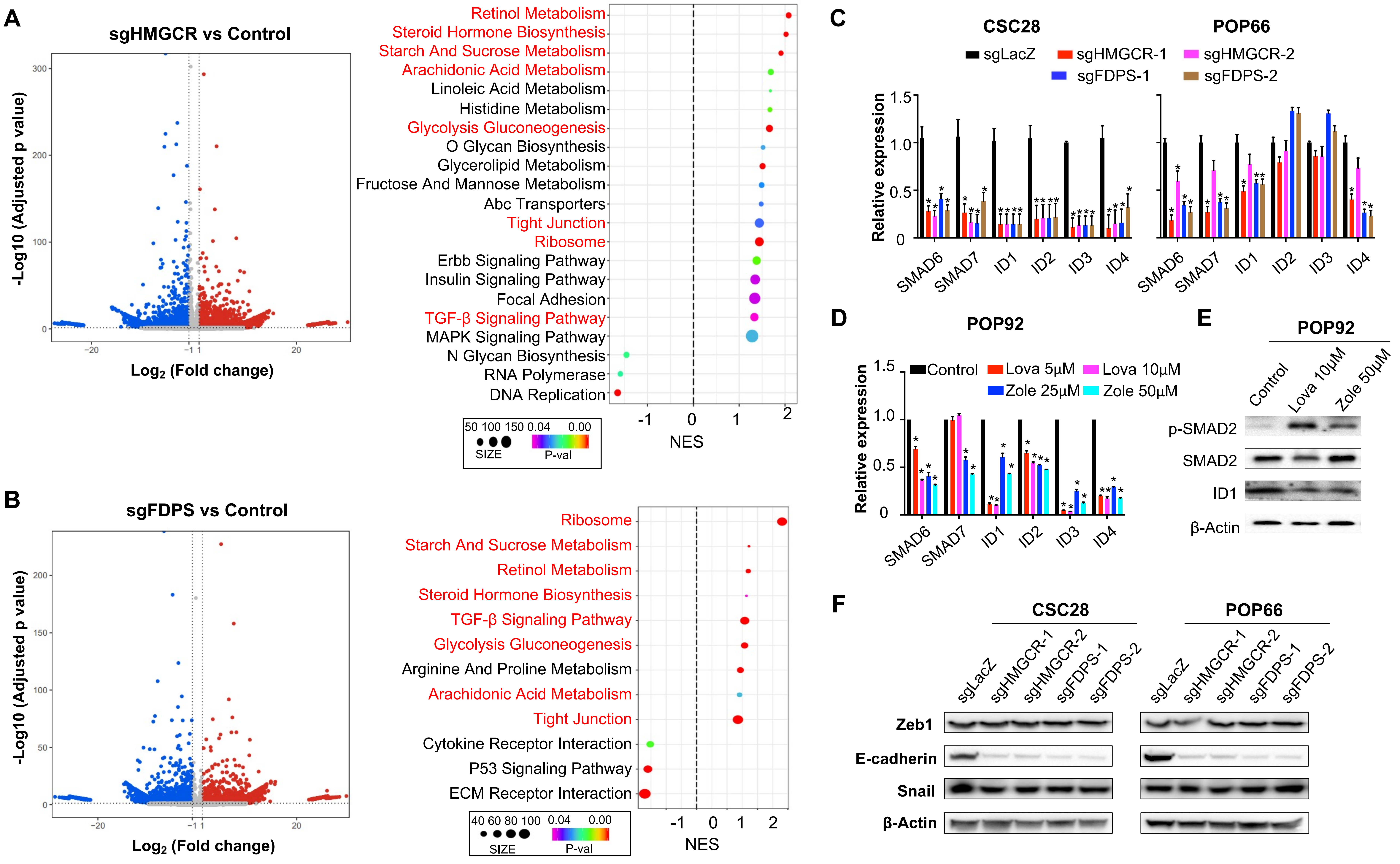

Supplementary Figure 6. RNA-seq analysis shows significantly dysregulated gene expression profiles after depletion of HMGCR or FDPS. A-B, Volcano plots and gene set enrichment analysis of significantly altered genes after HMGCR or FDPS knockout in colon CSC-enriched spheroid POP92. Common altered pathways are shown in RED. **C,** Significantly downregulated TGF-β signaling pathway members after HMGCR or FDPS knockout are validated in another two colon spheroid models, CSC28 and POP66. **D-E,** TGF-β signaling members were checked after lovastatin/zoledronate acid treatment at indicated concentrations in POP92 by real time PCR and western blot. **F,** The effect of HMGCR or FDPS depletion on EMT markers were investigated in CSC28 and POP66 by western blot. **P* < 0.05. Error bar, mean ± SD.

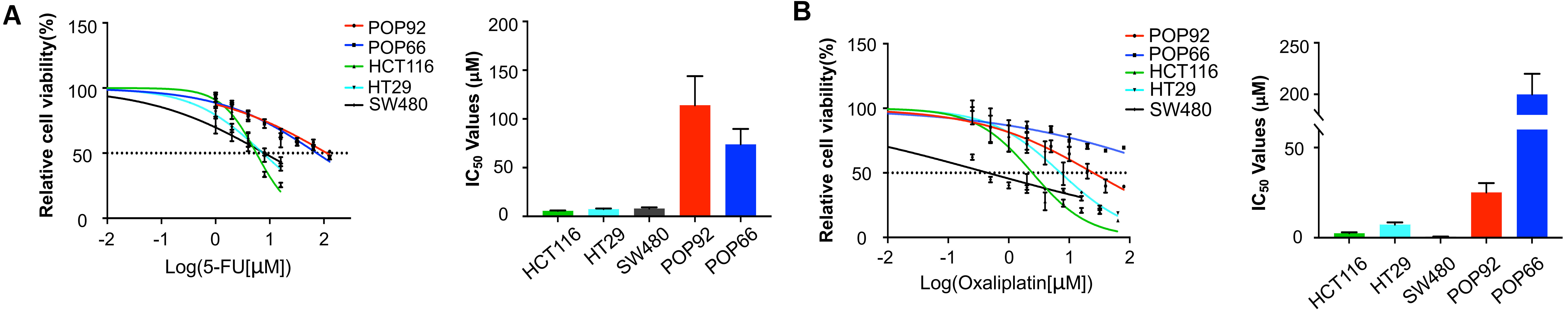

Supplementary Figure 7. Cytotoxicity and IC50 values of (**A**) 5-FU and (**B**) oxaliplatin in colon CSC-enriched spheroids and 2D long-established cancer cell lines. **A-B,** 5-FU and oxaliplatin dose response curve and IC50 value for Colon spheroids and 2D cancer cell lines. These cells were exposed to a serial concentration of 5-FU or oxaliplatin for 72hours, and cell viability was measured using MTS-based proliferation assay. All values (left panels) are normalized to untreated control. Bar graphs (right panels) show the IC50 values from three independent experiments.

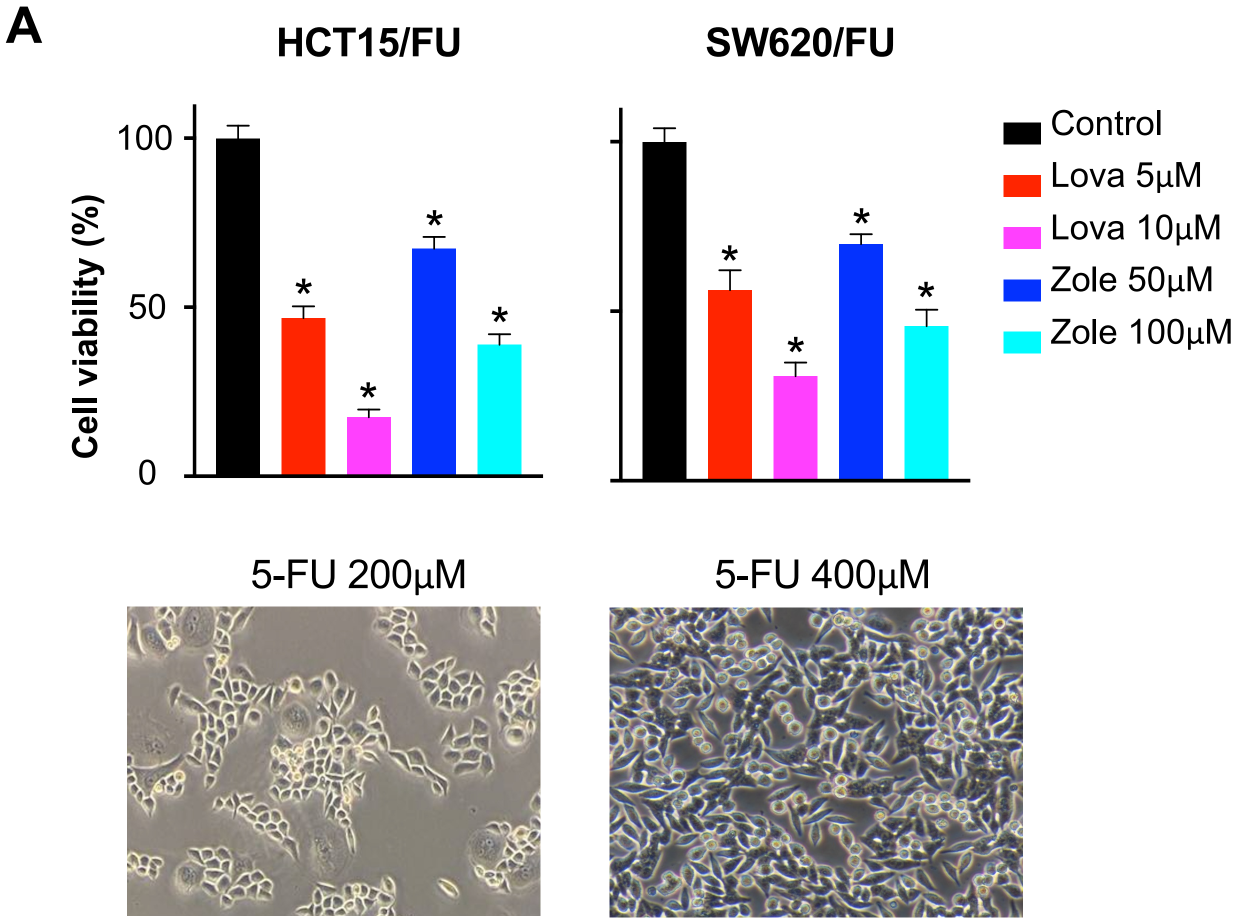

Supplementary Figure 8. The effect of HMGCR/FDPS blockade in drug-resistant colon cancer cells. A, HCT15/FU and SW620/FU are two laboratory-developed drug-resistant colon cancer cell lines that could survive under high concentrations of 5-FU (bottom panel). These cells were exposed to lovastatin or zoledronate acid for 72hours at indicated concentrations, and cell viability was measured using MTS-based proliferation assay. *$P < 0.05$. Error bar, mean ± SD.

Table S1. Essential gene list in POP92

Gene symbol	neg|score	neg|p-value	neg|fdr	neg|lfc	Rank
GGPS1	8.19E-14	3.72E-06	0.000206	-3.625	1
ATP6V1B2	2.98E-12	3.72E-06	0.000206	-2.2321	2
FDPS	2.66E-11	3.72E-06	0.000206	-4.5637	3
ACTL6A	7.99E-11	3.72E-06	0.000206	-2.4272	4
METTL3	6.15E-10	3.72E-06	0.000206	-1.5625	5
HDAC3	7.10E-10	3.72E-06	0.000206	-3.0654	6
TRMT5	9.03E-10	3.72E-06	0.000206	-2.0763	7
TRRAP	1.22E-09	3.72E-06	0.000206	-3.4993	8
PRMT1	1.36E-09	3.72E-06	0.000206	-2.2341	9
PRMT5	4.20E-09	3.72E-06	0.000206	-3.2304	10
TUBG1	1.24E-08	3.72E-06	0.000206	-2.518	11
WDR92	1.30E-08	3.72E-06	0.000206	-2.5754	12
WDR5	1.99E-08	3.72E-06	0.000206	-3.9898	13
WDR75	7.89E-08	3.72E-06	0.000206	-2.302	14
WDR82	9.76E-08	3.72E-06	0.000206	-7.0553	15
MTOR	1.32E-07	3.72E-06	0.000206	-4.2736	16
OGT	1.58E-07	3.72E-06	0.000206	-1.8506	17
ENY2	1.61E-07	3.72E-06	0.000206	-1.2907	18
HCFC1	1.65E-07	3.72E-06	0.000206	-2.0616	19
DR1	1.74E-07	3.72E-06	0.000206	-1.7373	20

RPS8	2.02E-07	3.72E-06	0.000206	-2.4996	21
MAP2K1	2.64E-07	3.72E-06	0.000206	-2.5927	22
MED24	4.27E-07	3.72E-06	0.000206	-1.7484	23
PSMB1	8.65E-07	3.72E-06	0.000206	-4.0437	24
NOP2	9.05E-07	2.60E-05	0.001155	-1.4176	25
MEPCE	1.49E-06	1.12E-05	0.00053	-1.1304	26
SETD1A	1.80E-06	1.12E-05	0.00053	-1.891	27
RPL8	1.92E-06	1.12E-05	0.00053	-2.6888	28
ZNF335	2.04E-06	1.12E-05	0.00053	-2.2507	29
PHB	2.49E-06	2.60E-05	0.001155	-2.6704	30
UHRF1	2.67E-06	3.34E-05	0.001392	-2.1293	31
UBE2N	2.71E-06	3.34E-05	0.001392	-2.025	32
DNMT1	3.91E-06	4.83E-05	0.00195	-1.541	33
RUVBL2	4.84E-06	6.32E-05	0.002475	-6.5264	34
MAPK1	5.45E-06	7.06E-05	0.002613	-2.3673	35
VRK1	6.18E-06	7.80E-05	0.002736	-1.9042	36
SUDS3	6.79E-06	7.80E-05	0.002736	-1.2925	37
FTSJ3	7.07E-06	0.00011521	0.003336	-1.1099	38
TRMT61A	7.33E-06	7.06E-05	0.002613	-2.43	39
TBL1XR1	9.41E-06	9.29E-05	0.003173	-0.87532	40
RPL5	1.08E-05	0.00010035	0.00326	-5.3542	41
POLR1C	1.09E-05	0.00010035	0.00326	-3.9816	42
METAP2	1.10E-05	0.00010778	0.003263	-2.2804	43

TUBB4B	1.11E-05	0.00010778	0.003263	-2.1272	44
CDK4	1.11E-05	0.00010778	0.003263	-1.1017	45
KAT8	1.27E-05	0.00011521	0.003336	-2.0421	46
PSMB5	1.48E-05	0.00012265	0.003476	-2.3639	47
WDR74	1.58E-05	0.00013008	0.00361	-2.7513	48
ATM	1.63E-05	0.00013751	0.003738	-0.80541	49
RRM2	1.85E-05	0.00016725	0.004455	-2.6504	50
POLE	2.10E-05	0.00019698	0.005046	-3.0442	51
RPL6	2.28E-05	0.00021928	0.005511	-4.3612	52
METTL1	2.38E-05	0.00017468	0.004562	-1.909	53
KMT2A	2.39E-05	0.00023415	0.005671	-1.0084	54
WDR61	2.51E-05	0.00023415	0.005671	-1.4804	55
IGF1R	2.64E-05	0.00024158	0.005719	-0.91329	56
RTF1	2.90E-05	0.00024901	0.005719	-1.3564	57
RNF20	2.92E-05	0.00024901	0.005719	-1.0131	58
RPL4	3.55E-05	0.00027874	0.006293	-3.953	59
FBL	3.83E-05	0.00030848	0.006848	-2.1528	60
TOP1	4.35E-05	0.00036794	0.008034	-1.823	61
TADA2B	4.57E-05	0.00038281	0.008094	-0.5818	62
CCND1	4.64E-05	0.00038281	0.008094	-2.5805	63
POLE2	5.34E-05	0.00042741	0.008895	-2.1308	64
MCRS1	6.20E-05	0.00050174	0.010282	-1.8205	65
ATP1A1	6.42E-05	0.00052404	0.010576	-2.4456	66

HMGCR	6.95E-05	0.0005612	0.011157	-1.3961	67
CHEK1	7.39E-05	0.00059837	0.011721	-1.5795	68
RPL3	8.33E-05	0.00062067	0.011923	-3.364	69
TAF1	8.48E-05	0.00063554	0.011923	-1.3093	70
GART	8.56E-05	0.00063554	0.011923	-1.2212	71
PARP1	8.85E-05	0.00066527	0.012307	-1.3425	72
YEATS4	9.95E-05	0.0007619	0.013902	-1.3326	73
RNF8	0.000108	0.00084367	0.015116	-1.2719	74
U2AF1	0.00011	0.0008511	0.015116	-2.6523	75
GTF3C4	0.000117	0.00088826	0.015568	-2.0085	76
RBBP5	0.000119	0.00090313	0.015623	-1.5446	77
CYP51A1	0.000132	0.0010146	0.017327	-1.0502	78
YTHDC1	0.000145	0.0011336	0.019113	-1.1668	79
FASN	0.000161	0.0012153	0.020235	-1.5361	80
RNF40	0.000171	0.0012822	0.021085	-1.1318	81
NAA50	0.000186	0.0013789	0.022218	-1.0441	82
IMPDH2	0.000188	0.0013937	0.022218	-1.1575	83
ADAR	0.00019	0.0014012	0.022218	-0.93688	84
POLA1	0.000193	0.0014235	0.022306	-4.0768	85
RARA	0.00024	0.0016985	0.026307	-0.95344	86
SIN3A	0.000294	0.0020478	0.031353	-1.1057	87
RRM1	0.000305	0.0021147	0.03165	-2.8202	88
BRCA2	0.000306	0.0021147	0.03165	-1.3287	89

TRMT1	0.000314	0.0029324	0.041553	-1.1257	90
KANSL1	0.000336	0.0022634	0.033498	-1.4671	91
DDB1	0.000346	0.00236	0.034545	-3.9629	92
HUWE1	0.000357	0.0024121	0.034923	-1.21	93
RUVBL1	0.000388	0.0026425	0.037847	-2.1882	94
KDM2A	0.000441	0.0056009	0.066611	-0.45738	95
RPL19	0.000483	0.0032594	0.045637	-2.0605	96
KANSL3	0.000488	0.0032892	0.045637	-0.91189	97
TUBB3	0.000501	0.0033932	0.046596	-0.57341	98
POLR2D	0.000538	0.0036608	0.04877	-2.0133	99
TGS1	0.000539	0.0046643	0.057527	-0.75819	100
AURKB	0.000545	0.003698	0.04877	-5.723	101
KDM6B	0.000545	0.003698	0.04877	-0.83016	102
KDM8	0.000546	0.003698	0.04877	-1.3535	103
ASH2L	0.000594	0.0040697	0.052725	-1.7937	104
CTR9	0.000595	0.0040771	0.052725	-1.0855	105
PAF1	0.000621	0.004196	0.053741	-1.0276	106
MSL1	0.00063	0.0042704	0.054173	-1.2478	107
VPRBP	0.000647	0.0043967	0.055249	-2.406	108
BAZ1B	0.000674	0.0045157	0.056214	-1.0805	109
TRMT10C	0.000736	0.004813	0.058816	-1.2062	110
TOP2A	0.000774	0.0051995	0.062662	-1.6545	111
POLE3	0.000778	0.0052218	0.062662	-0.85226	112

DMAP1	0.000886	0.0058388	0.068825	-1.0309	113
CDC73	0.000987	0.0063739	0.074475	-2.3532	114
MEN1	0.001027	0.0066861	0.077443	-0.66109	115
SAP130	0.001081	0.0069686	0.079335	-1.1705	116
CRTC2	0.001127	0.0072808	0.082187	-0.44147	117
KAT5	0.001234	0.008039	0.089983	-1.3008	118
BRAF	0.001336	0.0085444	0.094632	-2.4103	119
PSMD2	0.001346	0.0085965	0.094632	-1.0813	120
TRMT112	0.001566	0.0068125	0.078226	-3.5778	121

Table S2. Essential gene list in POP66

Gene symbol	neg|score	neg|p-value	neg|fdr	neg|lfc	Rank
ATP6V1B2	1.80E-09	3.72E-06	0.002475	-2.3856	1
WDR75	5.28E-07	3.72E-06	0.002475	-2.182	2
FTSJ3	1.60E-06	3.34E-05	0.014851	-1.2477	3
RPL19	5.80E-06	7.06E-05	0.020792	-1.8386	4
USP7	6.06E-06	7.80E-05	0.020792	-2.0039	5
DDB1	1.68E-05	0.000152	0.028465	-5.2297	6
METAP2	1.95E-05	0.000182	0.028465	-1.3984	7
POLR1C	2.27E-05	0.000212	0.028465	-2.6984	8
ACTL6A	2.56E-05	0.000242	0.028465	-1.5508	9
HCFC1	2.74E-05	0.000242	0.028465	-1.7677	10
KMT2D	2.89E-05	0.000249	0.028465	-1.9487	11
METTL3	2.51E-05	0.000256	0.028465	-1.6249	12
HDAC3	4.02E-05	0.000346	0.029994	-1.859	13
RPL3	4.10E-05	0.000361	0.029994	-2.3517	14
FDPS	4.13E-05	0.000361	0.029994	-2.0624	15
TUBG1	4.58E-05	0.000383	0.029994	-1.3062	16
SFPQ	4.70E-05	0.000383	0.029994	-1.7278	17
SYT2	5.37E-05	0.000427	0.030485	-0.73285	18
WDR82	5.40E-05	0.000435	0.030485	-1.8431	19
DMAP1	7.47E-05	0.000598	0.039851	-1.9869	20
ELP3	7.93E-05	0.000688	0.043612	-0.75907	21

NOP2	8.60E-05	0.000769	0.04658	-0.67645	22
DR1	0.000105	0.000836	0.048429	-1.5218	23
WDR74	0.000114	0.000911	0.048588	-1.8993	24
HUWE1	0.000117	0.000948	0.048588	-1.9813	25
TRMT112	0.000191	0.000955	0.048588	-2.5866	26
NAA50	0.000121	0.000985	0.048588	-0.71458	27
PSMB2	0.000133	0.001089	0.051803	-2.029	28
POLA1	0.000217	0.001691	0.075535	-2.0811	29
PRMT5	0.000229	0.001728	0.075535	-1.3296	30
HMGCR	0.000234	0.001758	0.075535	-1.8368	31
AURKA	0.000251	0.001847	0.07615	-1.2696	32
PRMT2	0.000257	0.001914	0.07615	-1.5555	33
WDR5	0.000264	0.001944	0.07615	-1.728	34
RUVBL1	0.000304	0.002219	0.084441	-2.8686	35
FCGR1B	0.000351	0.002494	0.092272	-1.1902	36
RRM1	0.000398	0.002821	0.101552	-2.1904	37
RPL4	0.000415	0.002903	0.101746	-2.4361	38
RUVBL2	0.000449	0.003118	0.105817	-0.85612	39
RNF20	0.000463	0.003178	0.105817	-1.3613	40
CHEK1	0.000562	0.003921	0.127385	-1.2644	41
LDB1	0.000601	0.004196	0.133074	-0.89502	42
RTF1	0.000634	0.004382	0.135736	-1.152	43
TAF1	0.000647	0.004486	0.135801	-1.1977	44

FLT3	0.000672	0.00459	0.135864	-1.6651	45
PSMD2	0.000835	0.005623	0.162828	-1.587	46
U2AF1	0.000863	0.005772	0.163577	-1.5794	47
RNF168	0.000968	0.006359	0.176465	-0.32033	48
BRAF	0.001043	0.00682	0.185391	-1.0245	49
GGPS1	0.00111	0.007229	0.192574	-1.0902	50
FBL	0.000956	0.007452	0.194307	-1.8517	51
TPK1	0.001162	0.007586	0.194307	-1.4432	52
KMT2C	0.001277	0.008374	0.208196	-0.90795	53
TUBB3	0.001291	0.00844	0.208196	-1.7602	54
RBBP5	0.00136	0.008872	0.214851	-1.3783	55
MGAM	0.001403	0.009176	0.218264	-0.90128	56
SUDS3	0.001434	0.009392	0.219472	-1.2521	57
PHB	0.001499	0.009771	0.22294	-0.70878	58
RPL8	0.001516	0.009875	0.22294	-1.2114	59

Table S3. Common essential genes for both colon CSC-enriched spheroids propagation *in vitro*

Gene symbol	POP92			POP66			Adj. Rank
	neg|p-value	neg|fdr	Rank	neg|p-value	neg|fdr	Rank	
ATP6V1B2	0.00000	0.0025	1	0.00000	0.0002	2	1
WDR75	0.00000	0.0025	2	0.00000	0.0002	14	2
ACTL6A	0.00024	0.0285	9	0.00000	0.0002	4	3
FDPS	0.00036	0.0300	15	0.00000	0.0002	3	4
GGPS1	0.00723	0.1926	50	0.00000	0.0002	1	5
METTL3	0.00026	0.0285	12	0.00000	0.0002	5	6
HDAC3	0.00035	0.0300	13	0.00000	0.0002	6	7
FTSJ3	0.00003	0.0149	3	0.00012	0.0033	38	8
TUBG1	0.00038	0.0300	16	0.00000	0.0002	11	9
HCFC1	0.00024	0.0285	10	0.00000	0.0002	19	10
WDR82	0.00044	0.0305	19	0.00000	0.0002	15	11
PRMT5	0.00173	0.0755	30	0.00000	0.0002	10	12
METAP2	0.00018	0.0285	7	0.00011	0.0033	43	13
POLR1C	0.00021	0.0285	8	0.00010	0.0033	42	14
RPL19	0.00007	0.0208	4	0.00326	0.0456	96	15
WDR5	0.00194	0.0762	34	0.00000	0.0002	13	16
DR1	0.00084	0.0484	23	0.00000	0.0002	20	17
NOP2	0.00077	0.0466	22	0.00003	0.0012	25	18

DDB1	0.00015	0.0285	6	0.00236	0.0345	92	19
RPL3	0.00036	0.0300	14	0.00062	0.0119	69	20
WDR74	0.00091	0.0486	24	0.00013	0.0036	48	21
RUVBL2	0.00312	0.1058	39	0.00006	0.0025	34	22
RPL8	0.00988	0.2229	59	0.00001	0.0005	28	23
PHB	0.00977	0.2229	58	0.00003	0.0012	30	24
HMGCR	0.00176	0.0755	31	0.00056	0.0112	67	25
SUDS3	0.00939	0.2195	57	0.00008	0.0027	37	26
NAA50	0.00099	0.0486	27	0.00138	0.0222	82	27
RPL4	0.00290	0.1017	38	0.00028	0.0063	59	28
DMAP1	0.00060	0.0399	20	0.00584	0.0688	113	29
RNF20	0.00318	0.1058	40	0.00025	0.0057	58	30
HUWE1	0.00095	0.0486	25	0.00241	0.0349	93	31
RTF1	0.00438	0.1357	43	0.00025	0.0057	57	32
POLA1	0.00169	0.0755	29	0.00142	0.0223	85	33
CHEK1	0.00392	0.1274	41	0.00060	0.0117	68	34
FBL	0.00745	0.1943	51	0.00031	0.0068	60	35
TAF1	0.00449	0.1358	44	0.00064	0.0119	70	36
TRMT112	0.00096	0.0486	26	0.00681	0.0782	121	37
RRM1	0.00282	0.1016	37	0.00211	0.0317	88	38
RUVBL1	0.00222	0.0844	35	0.00264	0.0378	94	39
U2AF1	0.00577	0.1636	47	0.00085	0.0151	75	40
RBBP5	0.00887	0.2149	55	0.00090	0.0156	77	41

TUBB3	0.00844	0.2082	54	0.00339	0.0466	98	42
PSMD2	0.00562	0.1628	46	0.00860	0.0946	120	43
BRAF	0.00682	0.1854	49	0.00854	0.0946	119	44

$Adj.\ Rank = \sqrt{Rank(POP92) * Rank\ (POP66)}$

Table S4. DNA sequences of primers for real time qPCR

Gene	Primer	Sequence (5'-3')
SREBP2	Forward	GGAGACCATGGAGACCCTCA
	Reverse	TTGCAGCATCTCGTCGATGT
HMGCR	Forward	GCCCTCAGTTCCAACTCACA
	Reverse	CAAGCTGACGTACCCCTGAC
HMGCS1	Forward	GGCTATAAAGCTGGTGGCGA
	Reverse	GGCATGGTGAAAGAGCAAGC
MVK	Forward	GCCCTCCGATACCATCAAGG
	Reverse	TCTCTGGGAACTTGAGCAGC
MVD	Forward	ATCAAGTACTGGGGCAAGCG
	Reverse	TTCAGCCAAATCCGGTCCTC
IDL1	Forward	CGGAGGCTGATCAGTGTTCTA
	Reverse	TGTTGCTTGTCGAGGTGGTT
FDPS	Forward	ATTTCCGCCCTTAGTGTGGG
	Reverse	GACAGGGGCATCCTGTTCC
SQLE	Forward	AGTTCGCCCTCTTCTCGGAT
	Reverse	GGTTCCTTTTCTGCGCCTCC
LSS	Forward	CTGTGCGTTCCATGGCCT
	Reverse	CACTGAAGTCCTGCCTGTGT
CYP51	Forward	GCAACCTCTTGTCCATGCTG
	Reverse	TATGGAGGACTTTTCACCCCTG
SC5D	Forward	GGTTGGTTAGCGAGTGCCC

	Reverse	GATCCATCACTTAGCCCCTGC
DHCR7	Forward	AAAGCCGCCCAGCTCTATAC
	Reverse	TACTTGTTCACAACCCCTGC
GGPPS1	Forward	GCCTGCGTGGACCGATTAG
	Reverse	ATGCCTGTGAAAGTTTGGTTCT
CD133	Forward	CCATAAAGCTGGACCCATTG
	Reverse	TTTTGGATTCATATGCCTTCTG
CD44	Forward	CCCAGATGGAGAAAGCTCTG
	Reverse	ACACCCCTGTGTTGTTTGCT
Lgr5	Forward	GCGGGAAACGCTCTGACATA
	Reverse	CATCCAGACGCAGGGATTGA
ALDH1A1	Forward	CAACAGAGGTTGGCAAGTTGA
	Reverse	ACCCCATGGTGTGCAAATTC
EphB2	Forward	GTGTGTAACAGAAGACGGGGG
	Reverse	TGGGGTCCTCGTAGGTGAAA
SMAD6	Forward	TTGCAACCCCTACCACTTCAGC
	Reverse	AGAATCGGACAGATCCAGTGGC
SMAD7	Forward	TTGCCTCGGACAGCTCAATT
	Reverse	TGCTGCGGTTGTAAACCCA
ID1	Forward	AATCCGAAGTTGGAACCCCC
	Reverse	GGAACGCATGCCGCCT
ID2	Forward	TGAAAGCCTTCAGTCCCGTG
	Reverse	TGAGCTTGGAGTAGCAGTCG

ID3	Forward	AGCGCGTCATCGACTACATT
	Reverse	TGACAAGTTCCGGAGTGAGC
ID4	Forward	AGCTCCGAAGGGAGTGACTA
	Reverse	TCGCTCTGGGTTTTACGAGG

Table S5. sgRNA sequences used in this study

Gene	sgRNA sequence
HMGCR-sg1-F	TGGAAGTAAATATACAGGA
HMGCR-sg1-R	TCCTGTATATTTACTTCCA
HMGCR-sg2-F	ATACTGTGTAGCTTGGTGG
HMGCR-sg2-R	CCACCAAGCTACACAGTAT
FDPS-sg1-F	TTGGAGGCAAGTATAACCG
FDPS-sg1-R	CGGTTATACTTGCCTCCAA
FDPS-sg2-F	GGATTCATCCCTTACCCGC
FDPS-sg2-R	GCGGGTAAGGGATGAATCC
sgLacZ-F	CACCGCCCGAATCTCTATCGTGCGG
sgLacZ-R	CCGCACGATAGAGATTCGGGCGGTG
METTL3-sg1-F	CACCGTGTGAAGCGTAGCACAGACG
METTL3-sg1-R	CGTCTGTGCTACGCTTCACACGGTG
METTL3-sg2-F	CACCGACCATCTTACCACTCTTCCA
METTL3-sg2-R	TGGAAGAGTGGTAAGATGGTCGGTG
